# Supplementary material for: Metabolite changes in blood predict the onset of tuberculosis
Source: Nat Commun. 2018 Dec 6;9:5208. doi: 10.1038/s41467-018-07635-7 (PMC6283869; doi:10.1038/s41467-018-07635-7)
Supplement: Supplementary file 1 — Supplementary Information [file 41467_2018_7635_MOESM1_ESM.pdf]

Metabolite changes in blood predict the onset of tuberculosis

Supplementary Information

The GC6 Consortium

2018-10-16

Supplementary Figures

Supplementary Figure 1. *Design of the GC6 study. Participants were recruited among household contacts of a TB index case in South Africa (SUN), The Gambia (MRC), Ethiopia (AHRI) and Uganda (MAK). Each participant was followed up for 2 years, and within that time, up to three samples have been collected. Individuals who developed TB within three months after enrollment or with concomitant diseases were excluded. Participants who eventually developed TB were considered progressors and compared to those who remained healthy during the follow-up.*

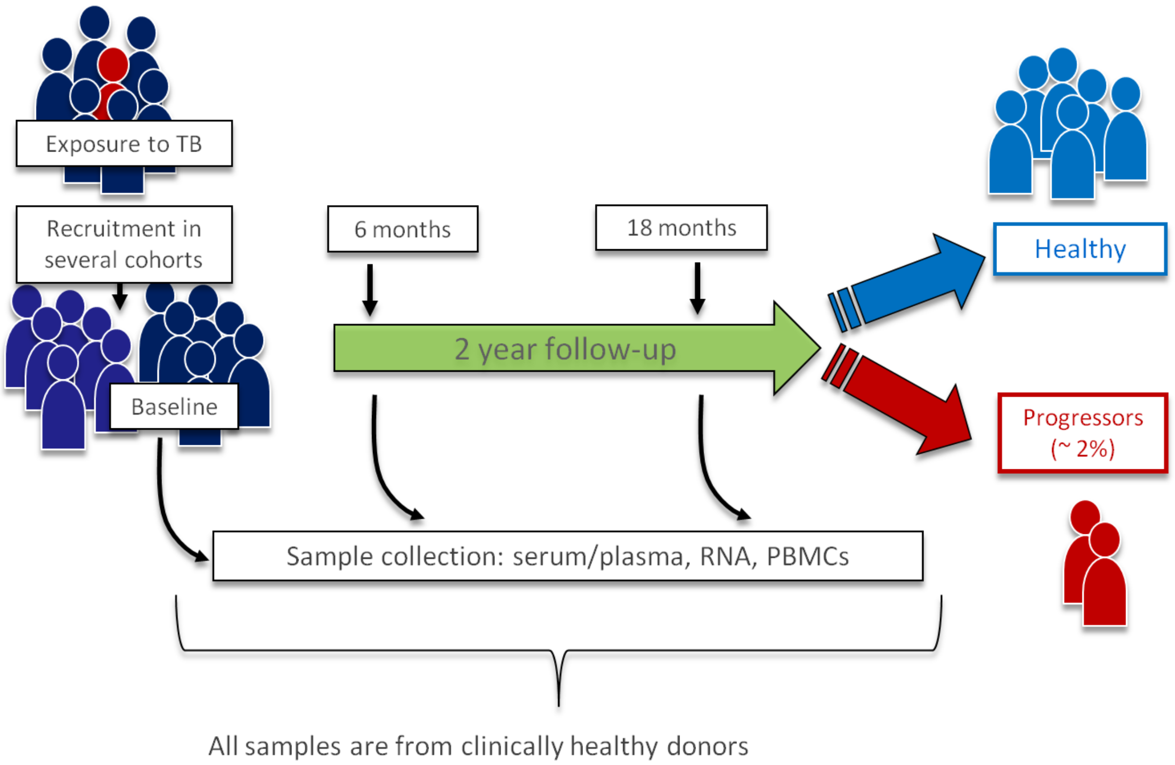

Supplementary Figure 2. *Validation scheme in the study.* ***A****, scheme of the validation using a blinded hold-out validation set. Training of the models was based on the unblinded training set, and selected models were applied blindly to the validation set.* ***B****, validation using external data sets from TB patients, healthy controls and patients sufferning from other diseases. GC6 progressor models were tested in the external data sets, and, vice versa, models based on external data sets were tested in the GC6 data set.*

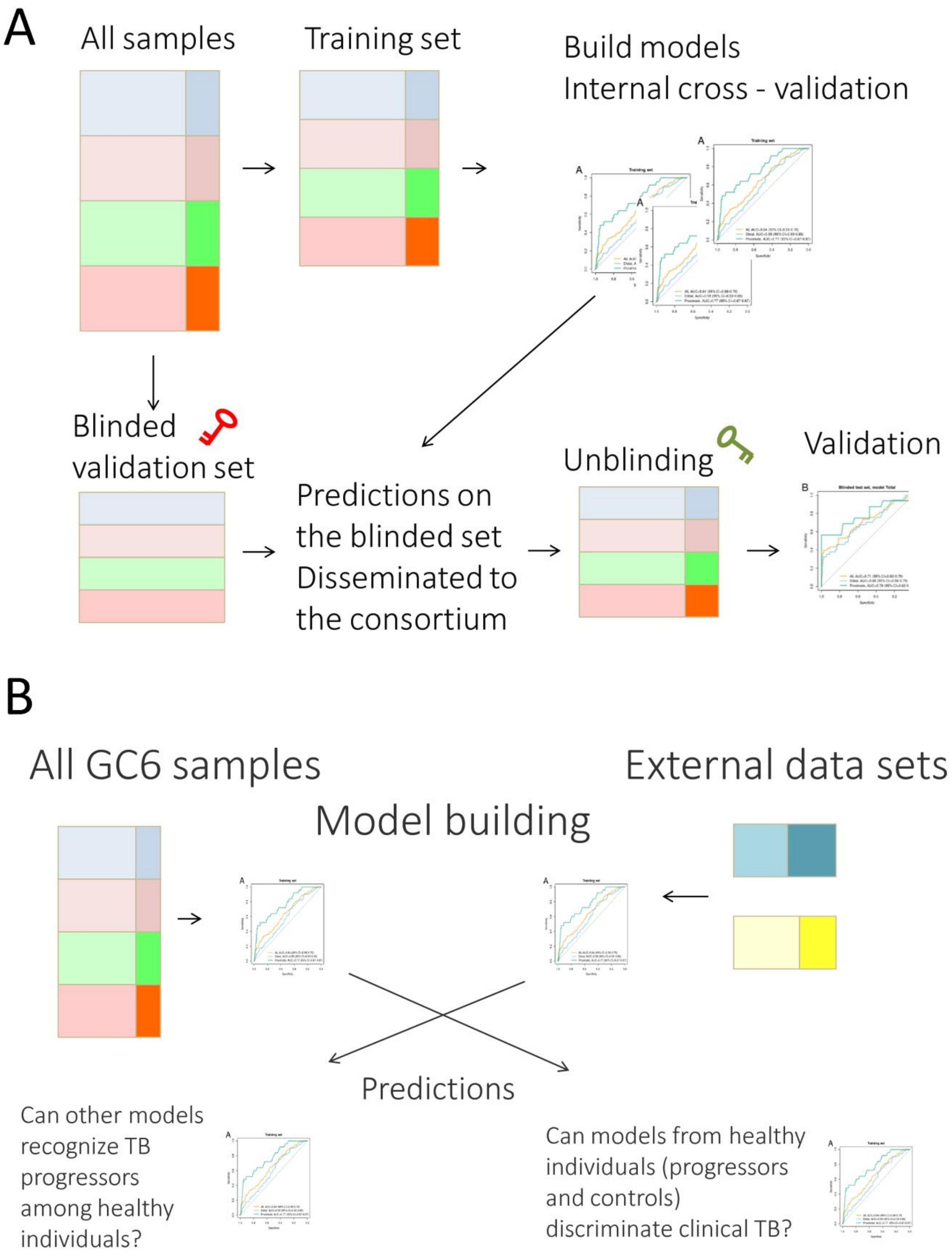
A
All samples
Training set
Build models
Internal cross - validation
Blinded
validation set
Predictions on
the blinded set
Disseminated to
the consortium
Unblinding
Validation
B
All GC6 samples
External data sets
Model building
Predictions
Can other models
recognize TB
progressors
among healthy
individuals?
Can models from healthy
individuals (progressors
and controls)
discriminate clinical TB?

Supplementary Figure 3. *Receiver-operator characteristic (ROC) curves showing the performance of all models in k-fold cross-validation within each training sample set and each stratum. All models where trained on the training data only and cross-validated using a 10-fold k-fold cross validation. TOT, total model trained on all samples from all cohorts; MRC, SUN, AHRI and MAK, models trained on samples from the respective sites; all, all samples; proximate, samples closer than 5 months to diagnosis; distal, samples further than 5 from diagnosis.*

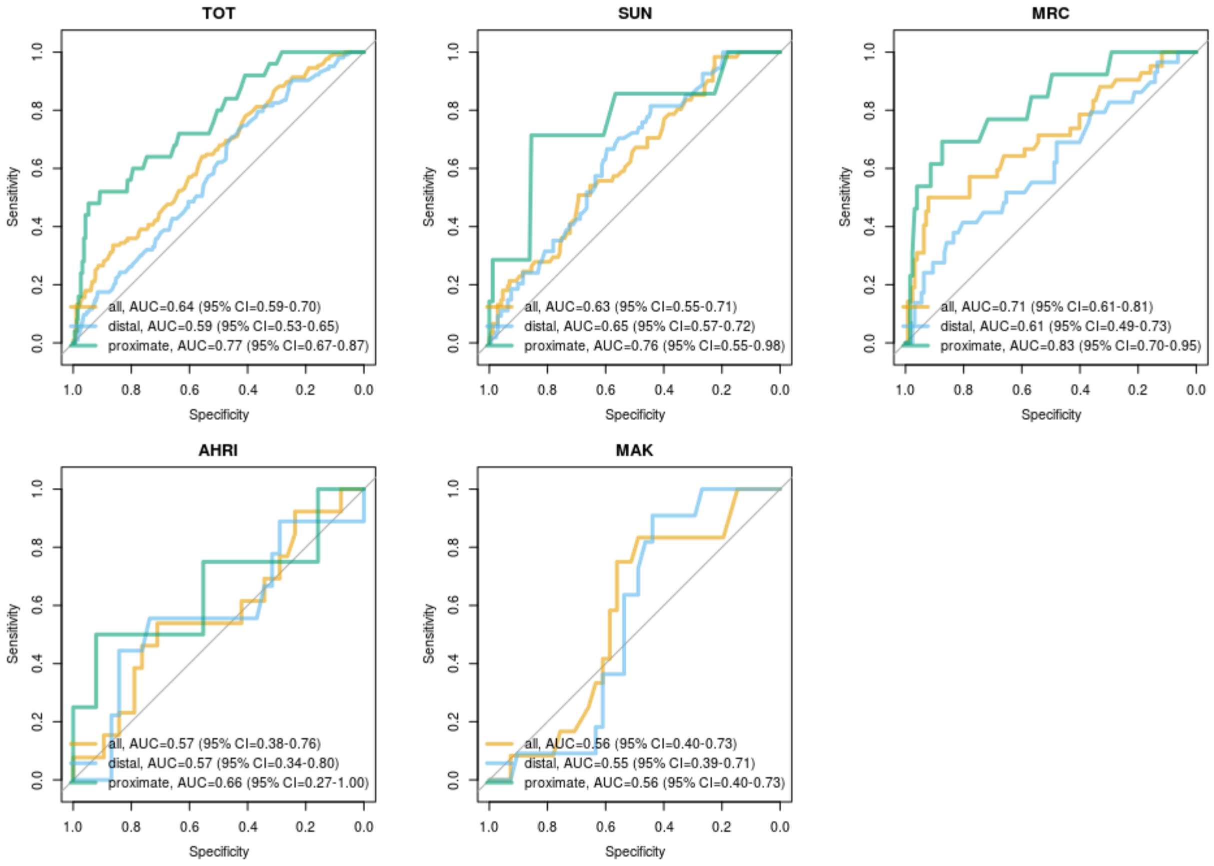

Supplementary Figure 4. *Cross-validation of the models between the different sites using training samples only. Each model was constructed using training set samples from a given site, and then validated on all samples for another site.*

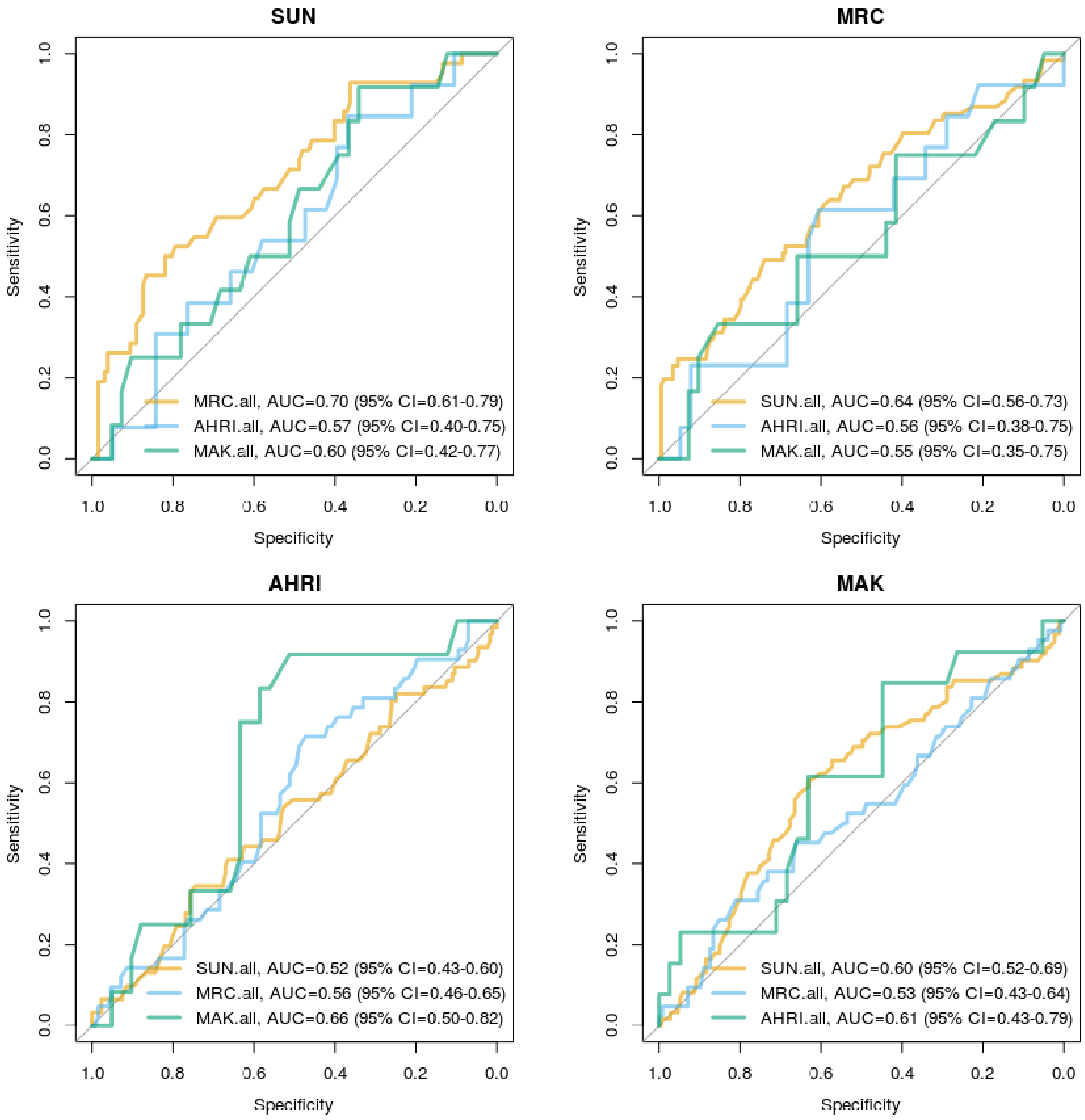

Supplementary Figure 5. *ROC curves showing the performance of predictions of models based on the GC6 cohort when applied to external data sets (TB-HEALTHY and TB-ORD).*

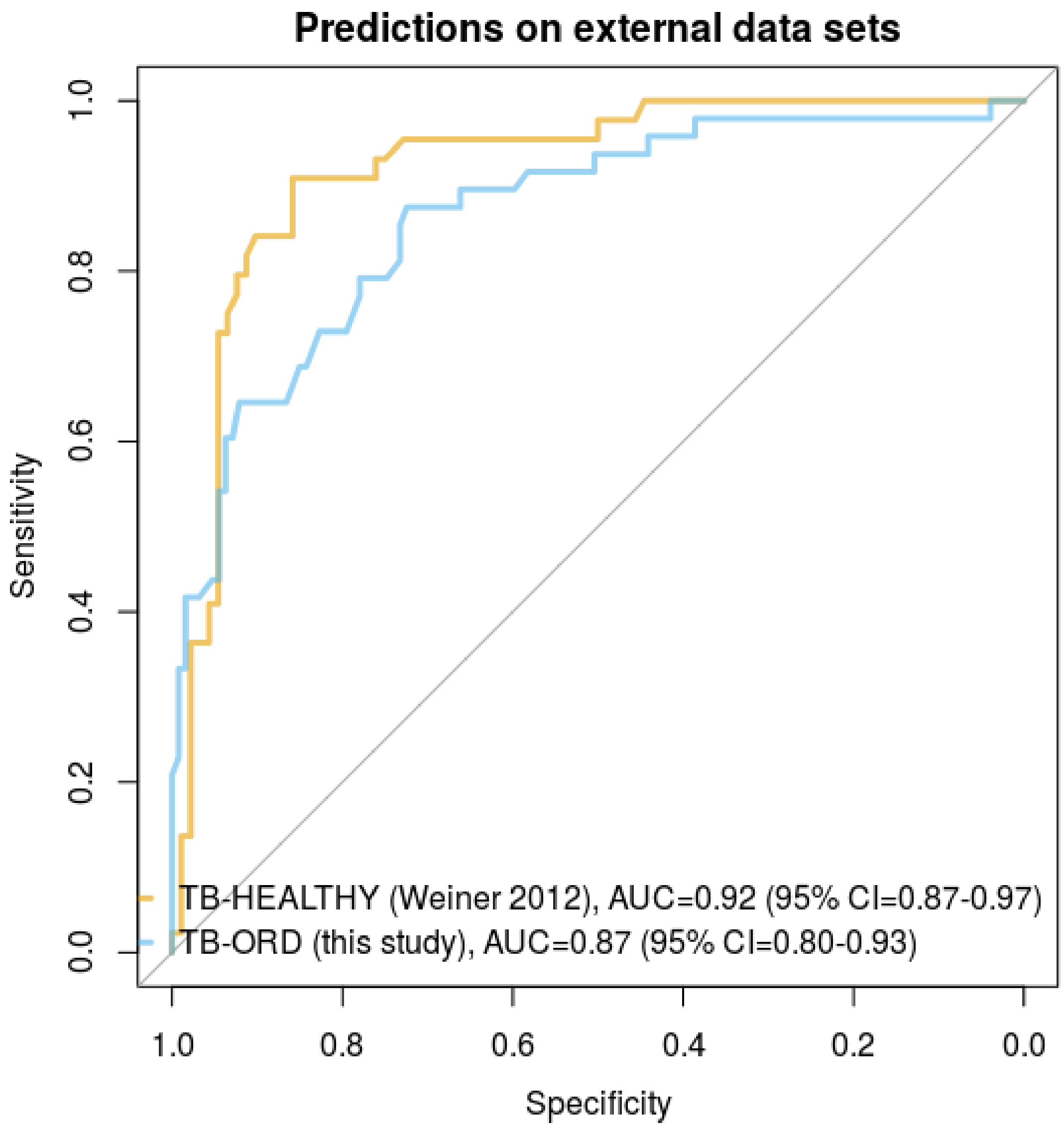

Supplementary Figure 6. Internal performance of models built on the TB-HEALTHY data set depending on the number of variables used. For each number of variables, 100 random forest models were built. AUC of the models is shown on the y axis, and number of variables on the x axis. Vertical line indicates the chosen number of variables for the reduced model.

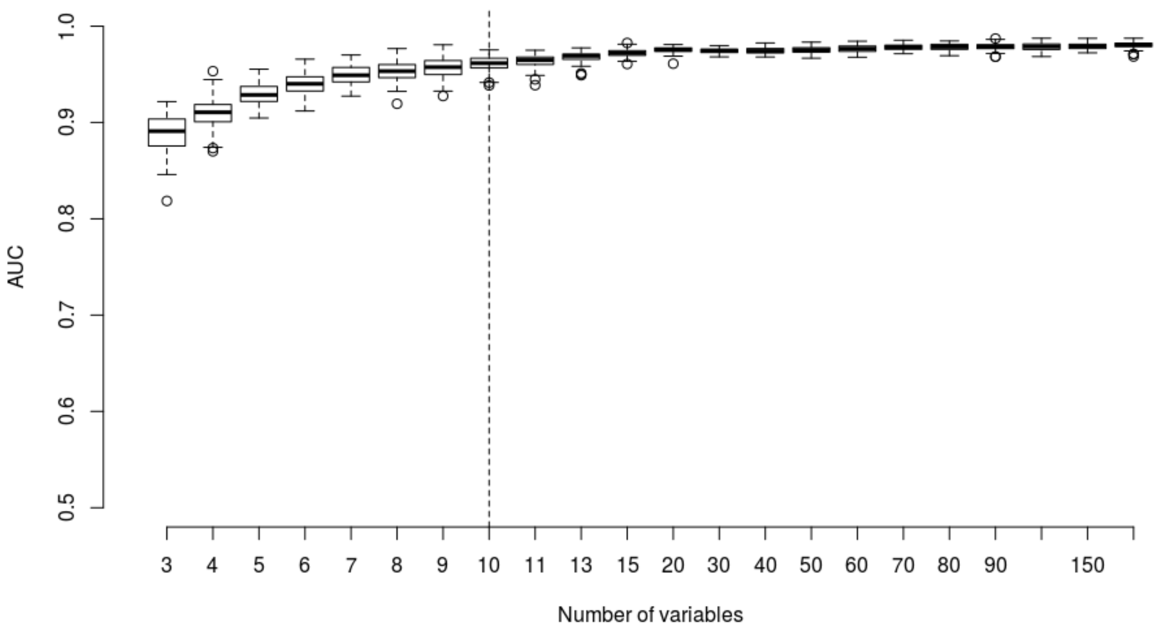

Supplementary Figure 7. *Normalized profiles of selected compounds and loess fits showing changes in compounds in progressors and controls. Horizontal axis – time to TB (for progressors) or time to study discharge (for controls); vertical axis – relative abundance. Shaded purple area indicates 95% confidence intervals. Solid green line indicates median for controls and dashed green lines indicate first and third quartiles for controls.*

A. M.32497: 10-undecenoate (11:1n1); B. M.37418: 1-pentadecanoyl-GPC (15:0); C. M.19324: 1-stearoyl-GPI (18:0); D. M.6146: 2-aminoadipate; E. M.32197: 3-(4-hydroxyphenyl)lactate; F. M.22053: 3-hydroxydecanoate;

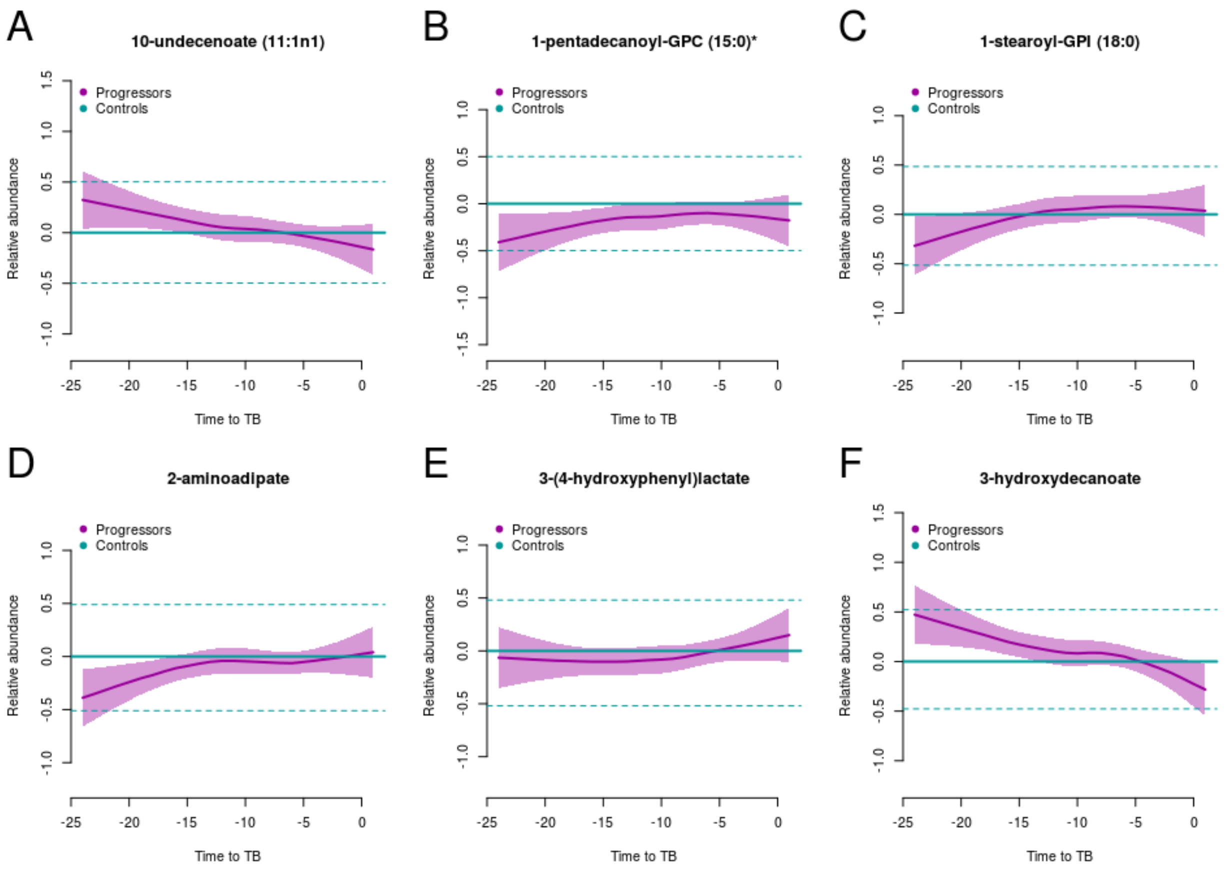

A. M.15681: 4-guanidinobutanoate; B. M.1559: 5,6-dihydrouracil; C. M.43231: 6-oxopiperidine-2-carboxylate; D. M.1107: allantoin; E. M.32586: bilirubin (E,E); F. M.43807: bilirubin (Z,Z);

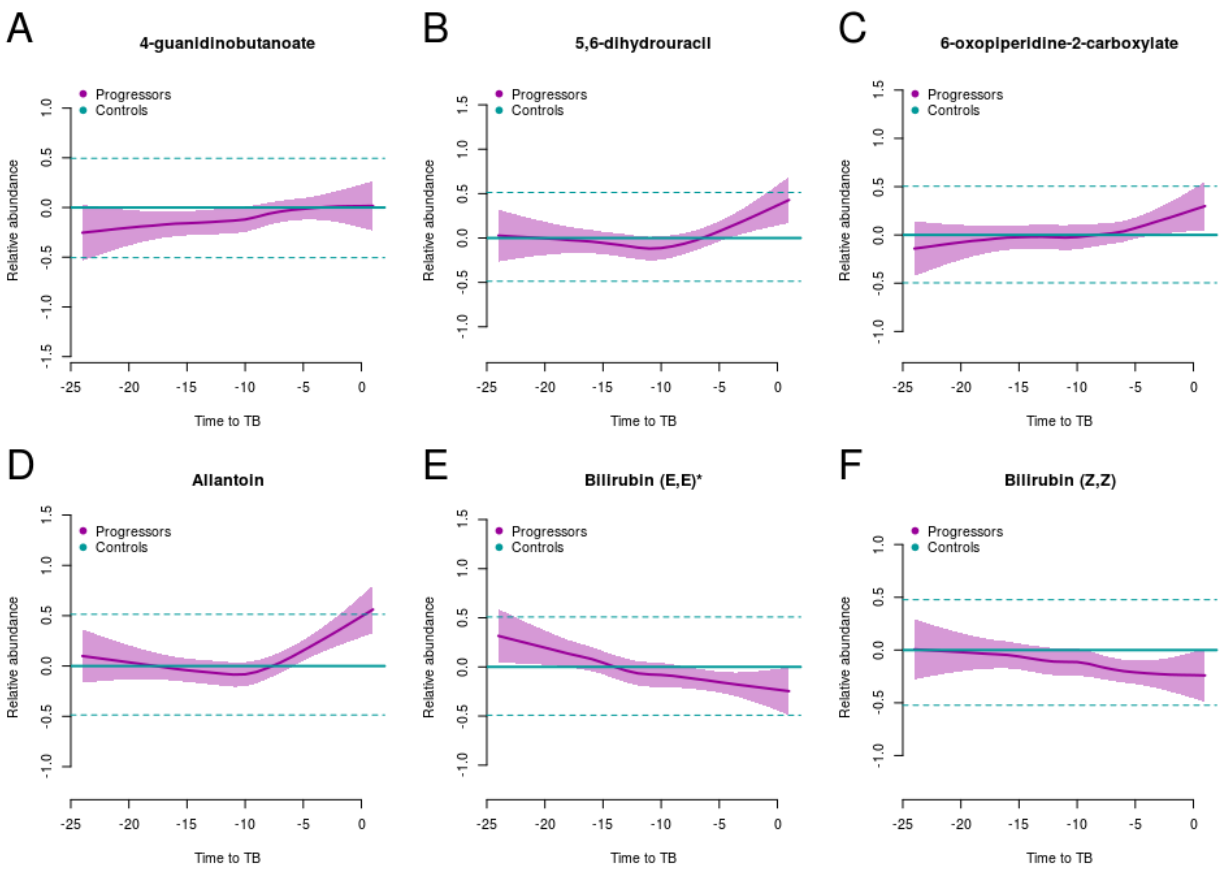

A. M.1712: cortisol; B. M.33941: decanoylcarnitine (C10); C. M.32425: dehydroisoandrosterone sulfate (DHEA-S); D. M.33973: epiandrosterone sulfate; E. M.33477: erythronate; F. M.34456: gamma-glutamylisoleucine;

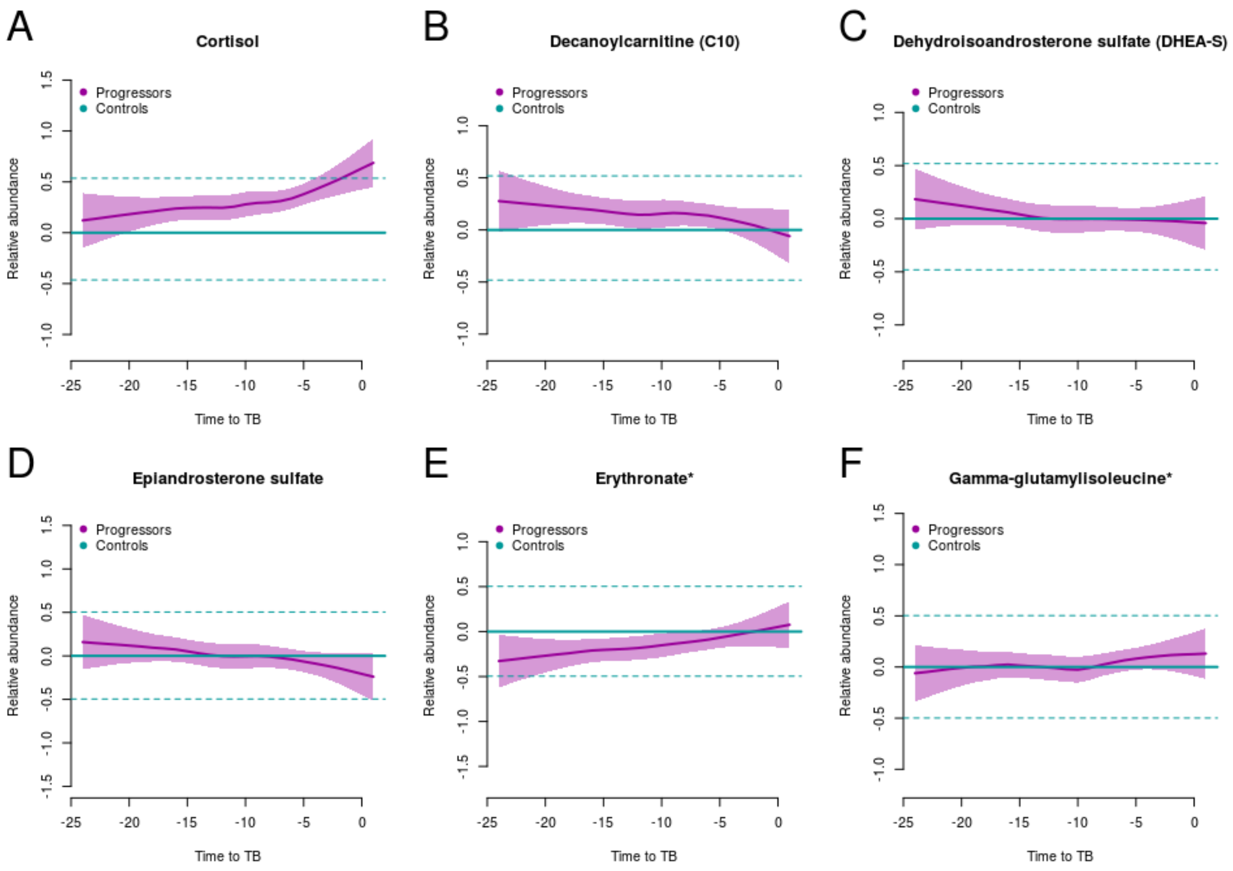

A. M.18369: gamma-glutamylleucine; B. M.33422: gamma-glutamylphenylalanine; C. M.2734: gamma-glutamyltyrosine; D. M.20488: glucose; E. M.15122: glycerol; F. M.32346: glycochenodeoxycholate;

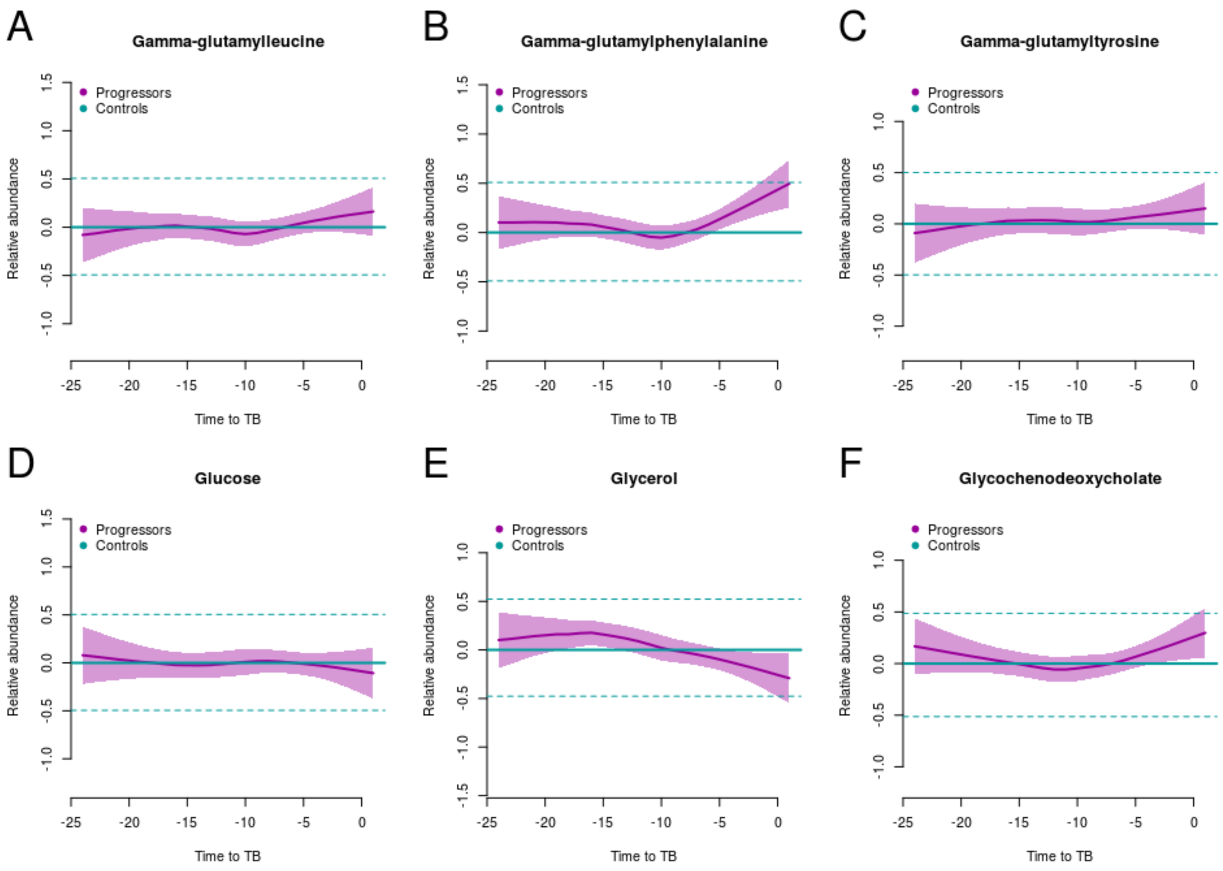

A. M.18476: glycocholate; B. M.18477: glycodeoxycholate; C. M.39379: glycoursodeoxycholate; D. M.43802: guanidinoacetate; E. M.32328: hexanoylcarnitine (C6); F. M.15140: kynurenine;

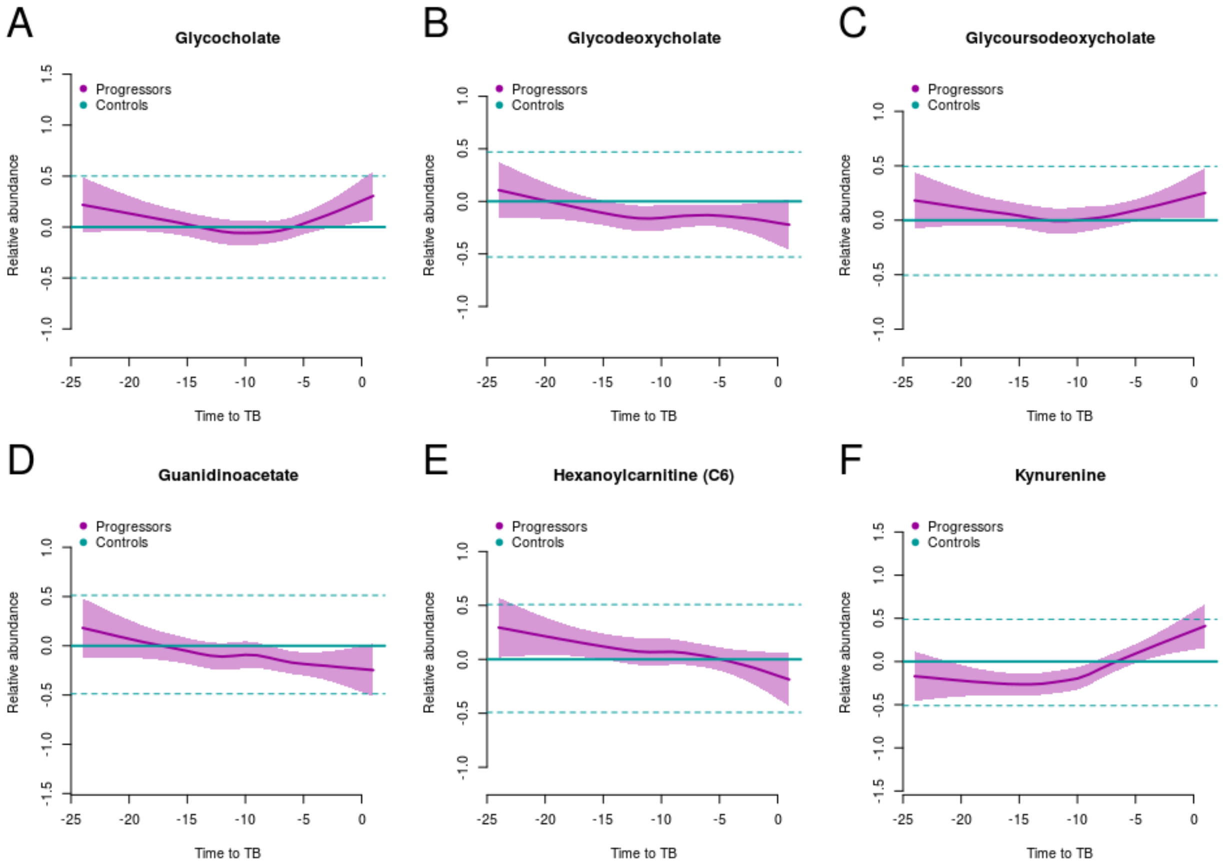

A. M.527: lactate; B. M.1645: laurate (12:0); C. M.60: leucine; D. M.1105: linoleate (18:2n6); E. M.34035: linolenate [alpha or gamma; (18:3n3 or 6)]; F. M.1365: myristate (14:0);

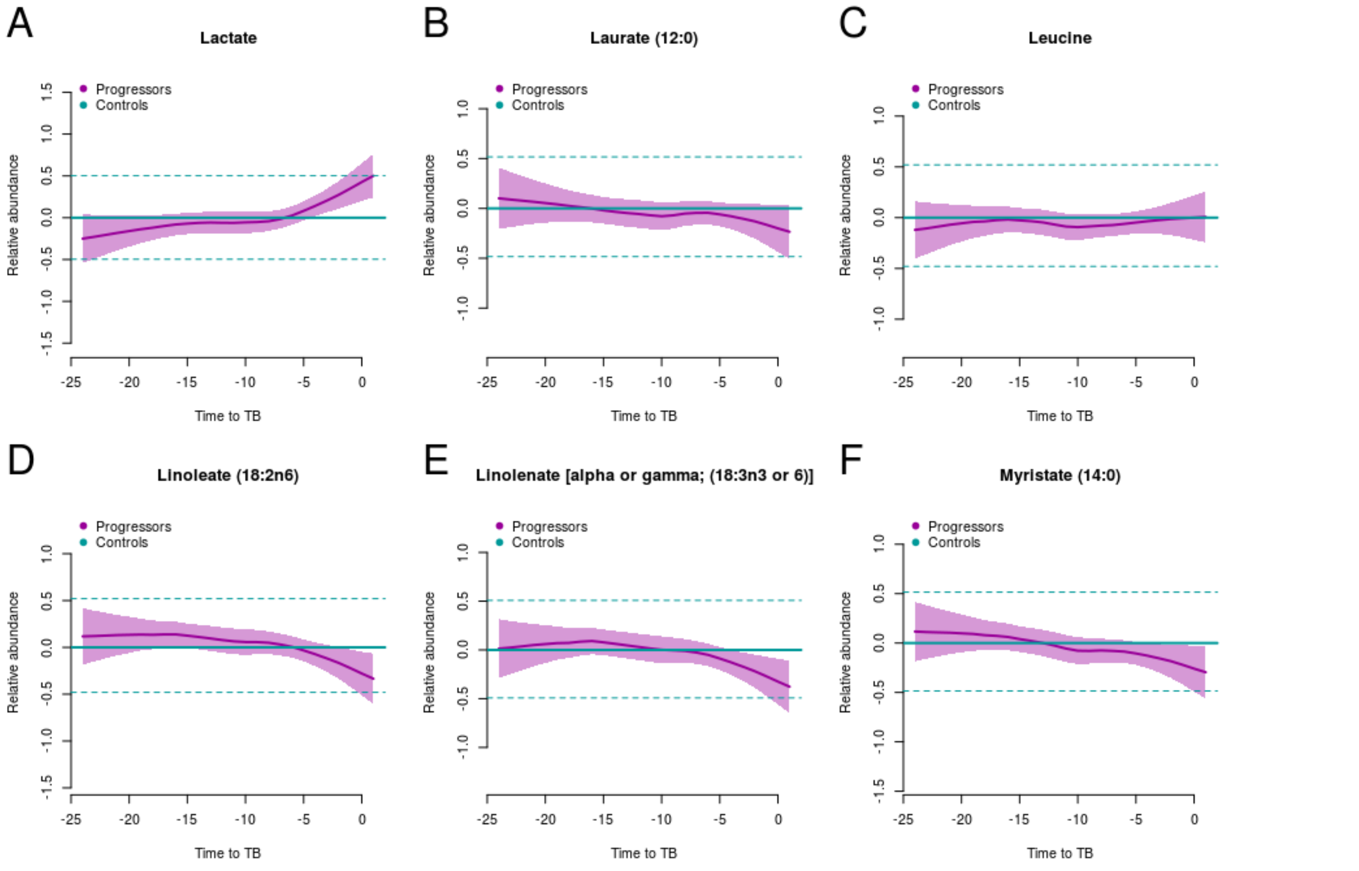

A. M.32418: myristoleate (14:1n5); B. M.35137: N2,N2-dimethylguanosine; C. M.36752: N6-acetyllysine; D. M.35157: N6-carbamoylthreonyladenosine; E. M.33967: N-acetylisoleucine; F. M.1587: N-acetylleucine;

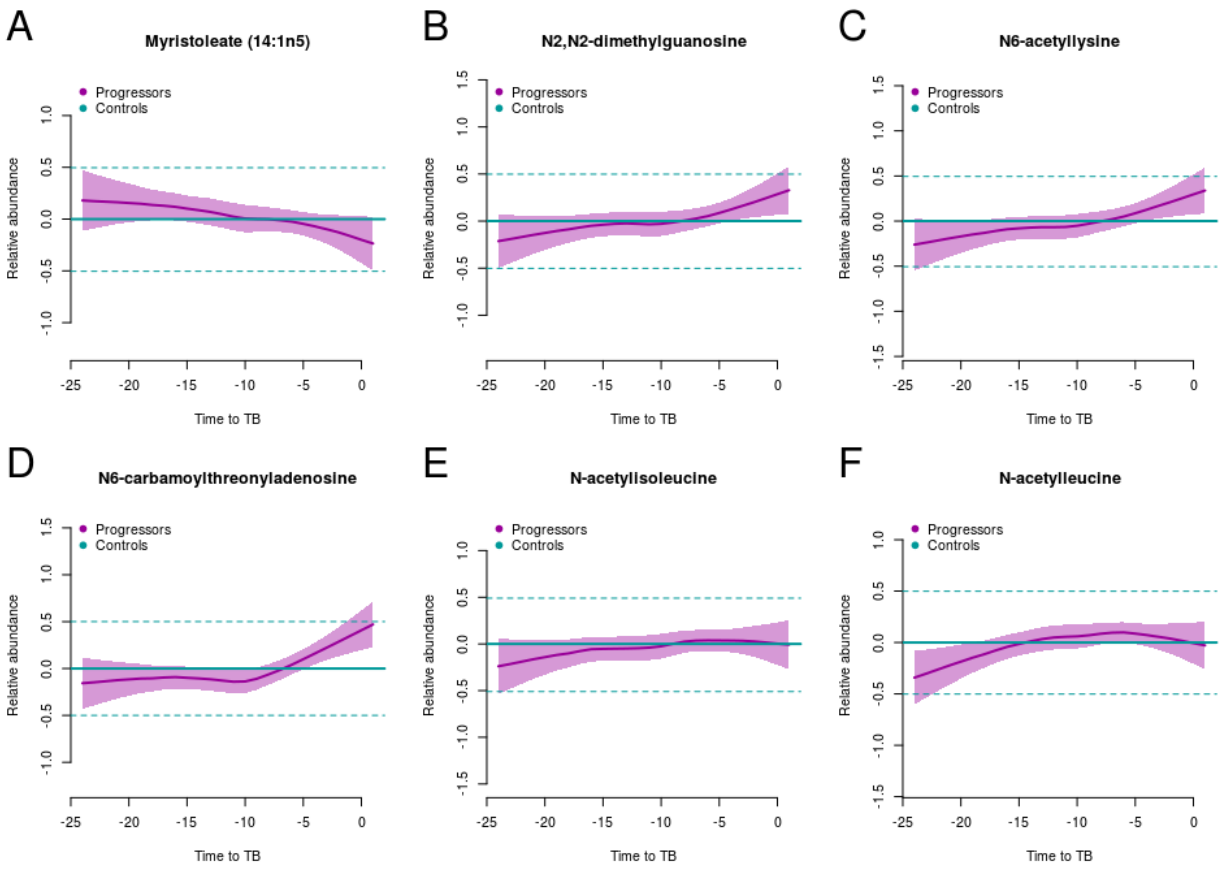

A. M.33950: N-acetylphenylalanine; B. M.33939: N-acetylthreonine; C. M.32390: N-acetyltyrosine; D. M.33936: octanoylcarnitine (C8); E. M.1336: palmitate (16:0); F. M.64: phenylalanine;

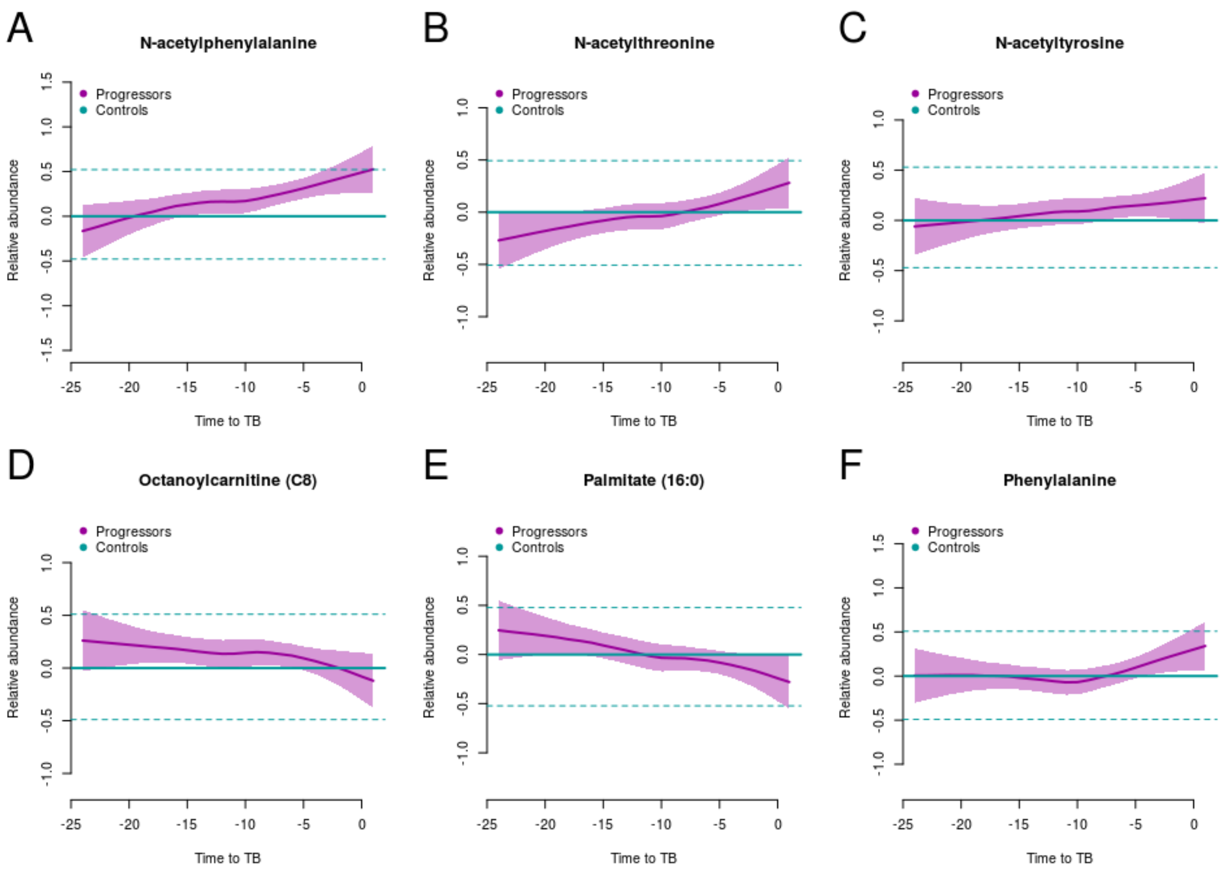

A. M.18494: taurochenodeoxycholate; B. M.18497: taurocholate; C. M.32807: taurocholenate sulfate; D. M.36850: taurolithocholate 3-sulfate; E. M.1299: tyrosine; F. M.15136: xanthosine;

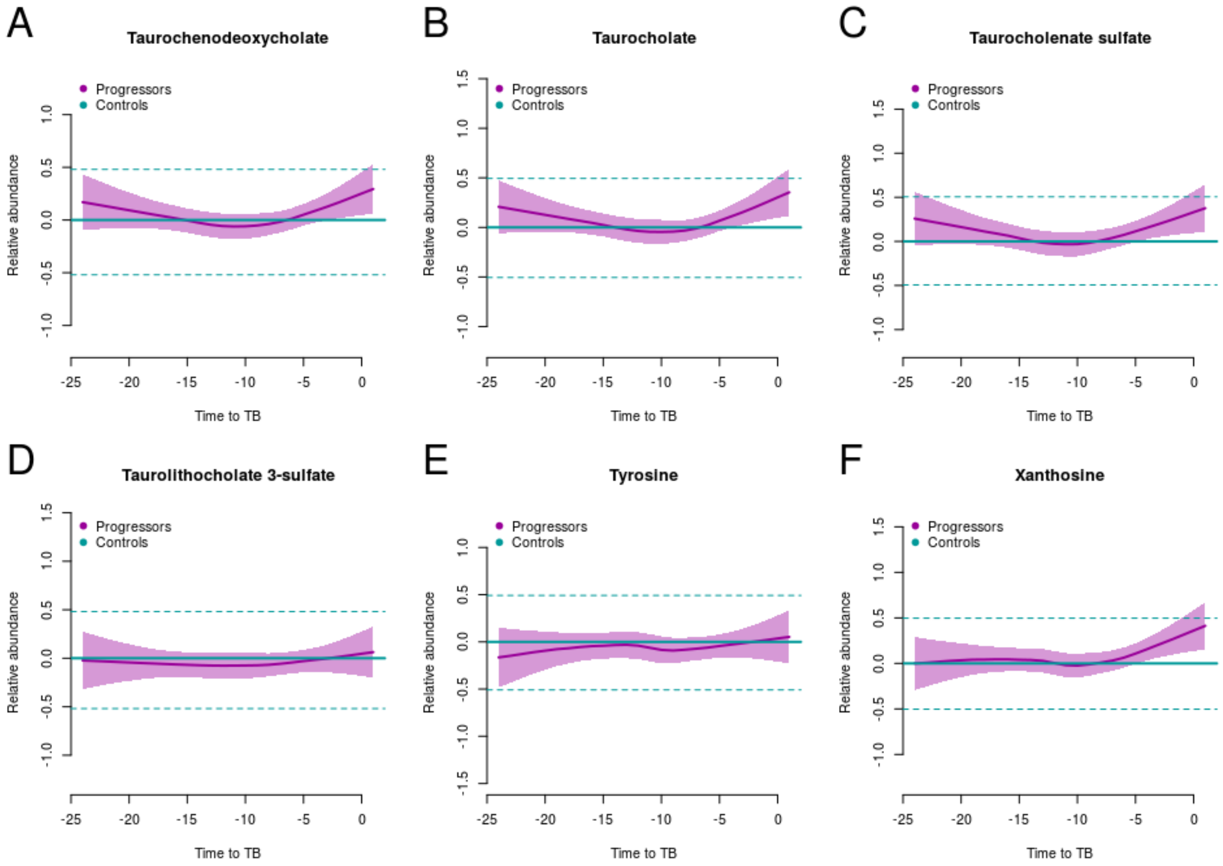

A. M.46500: 3-CMPFP; B. M.46590: X - 07765; C. M.47608: N6-succinyladenosine; D. M.46932: X - 12104; E. M.46618: X - 12442; F. M.46733: X - 13435;

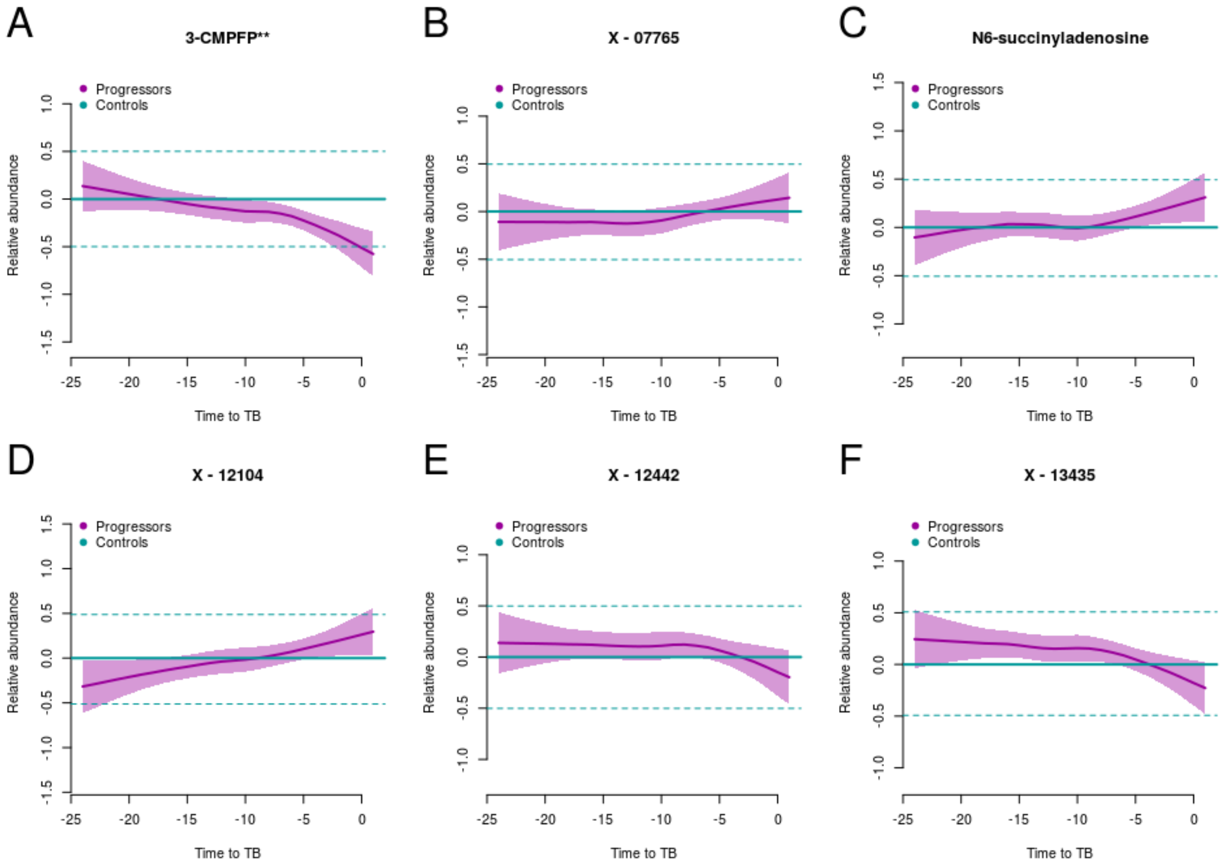

A. M.46661: X - 15245; B. M.46640: X - 15469; C. M.46473: 1-carboxyethylphenylalanine; D. M.46388: dodecadienoate (12:2); E. M.46398: X - 21353; F. M.46503: X - 21458;

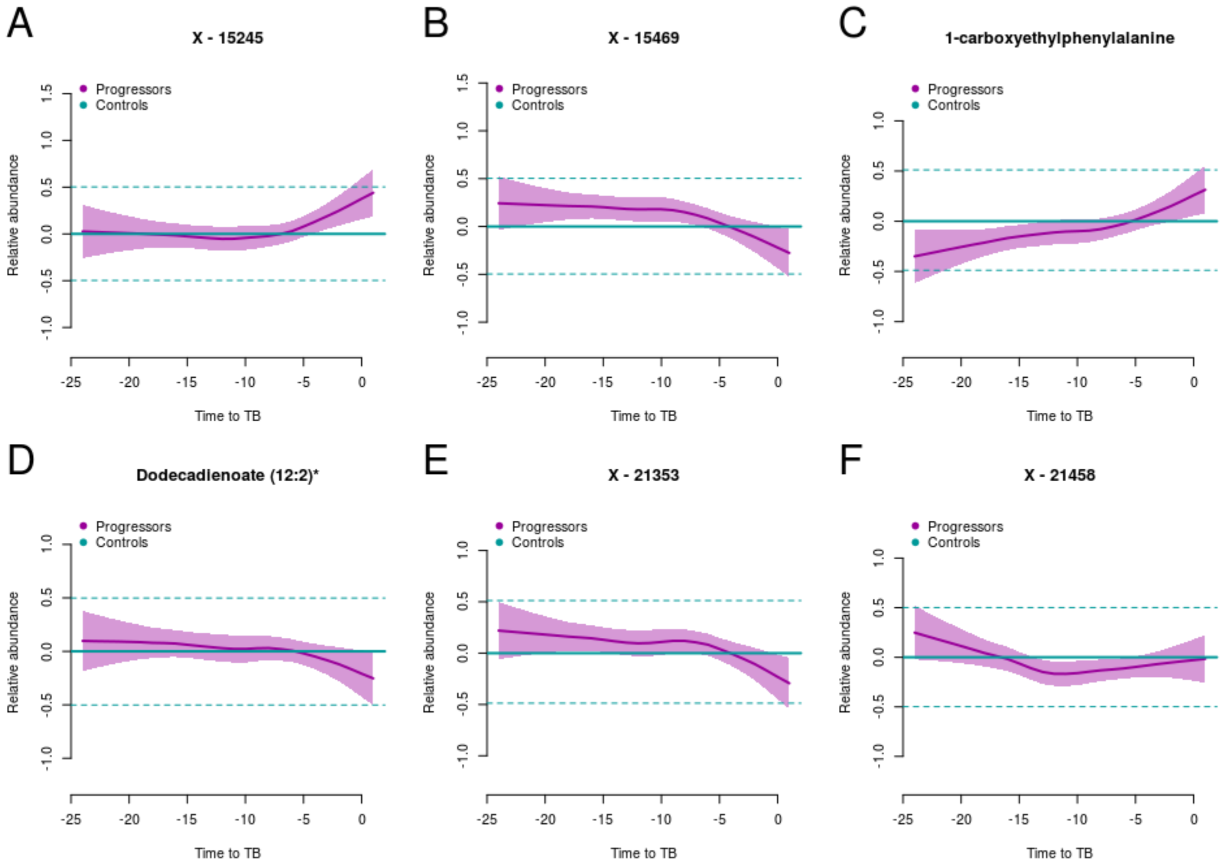

A. M.46674: X - 21607; B. M.47640: androsterone glucuronide; C. M.47783: X - 22519; D. M.38002: 1,2-propanediol; E. M.39609: 16-hydroxypalmitate; F. M.44630: 1-dihomo-linolenoyl-GPE (20:3n3 or 6);

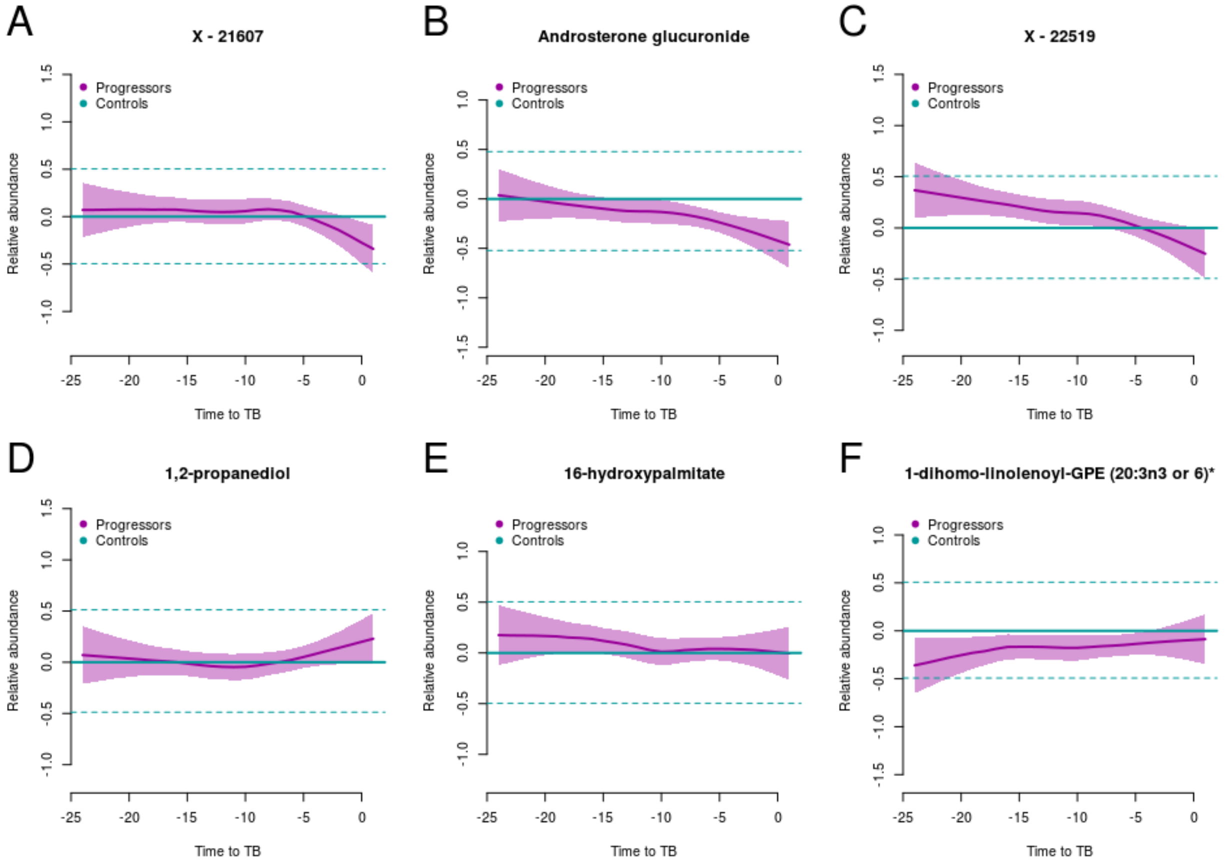

A. M.36594: 1-linoleoyl-GPI (18:2); B. M.44621: 1-(1-enyl-oleoyl)-GPE (P-18:1); C. M.36746: 2-hydroxy-3-methylvalerate; D. M.33173: 2-hydroxyacetaminophen sulfate; E. M.21044: 2-hydroxybutyrate (AHB); F. M.42489: 2-hydroxydecanoate;

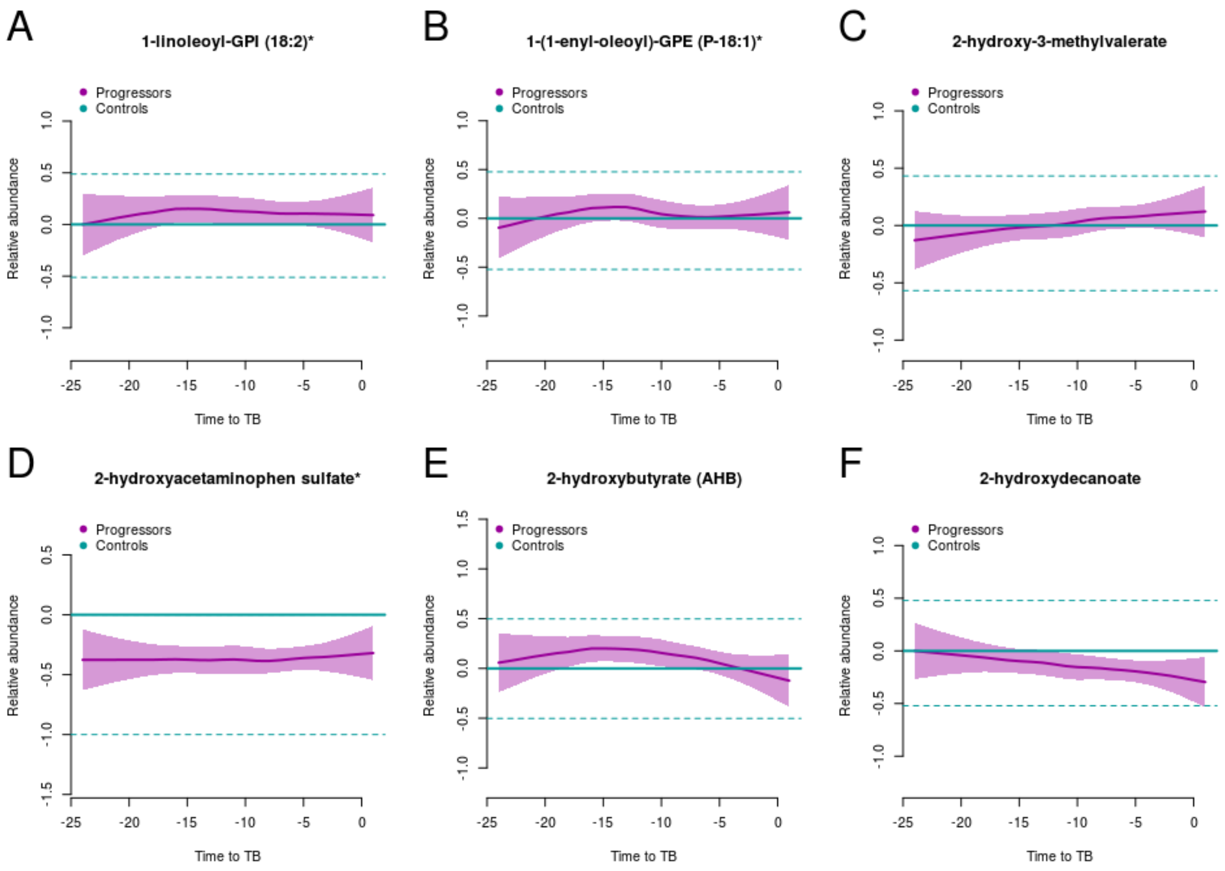

A. M.37253: 2-hydroxyglutarate; B. M.43400: 2-piperidinone; C. M.542: 3-hydroxybutyrate (BHBA); D. M.37202: androstenediol (3beta,17beta) disulfate (1); E. M.37187: 5alpha-androstan-3beta,17alpha-diol disulfate; F. M.37190: 5alpha-androstan-3beta,17beta-diol disulfate;

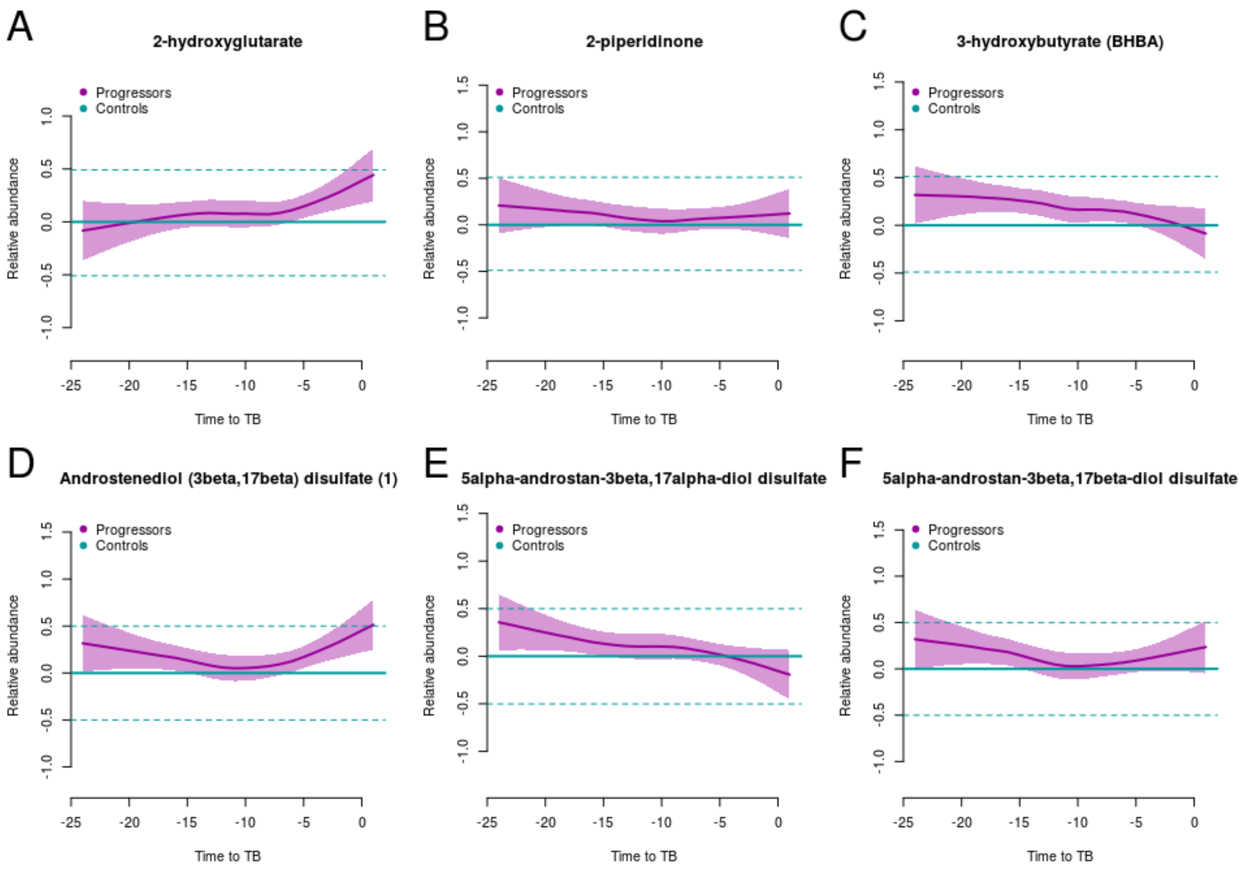

A. M.1126: alanine; B. M.12129: beta-hydroxyisovalerate; C. M.1564: citrate; D. M.1769: cortisone; E. M.553: cotinine; F. M.37104: cyclo(leu-pro);

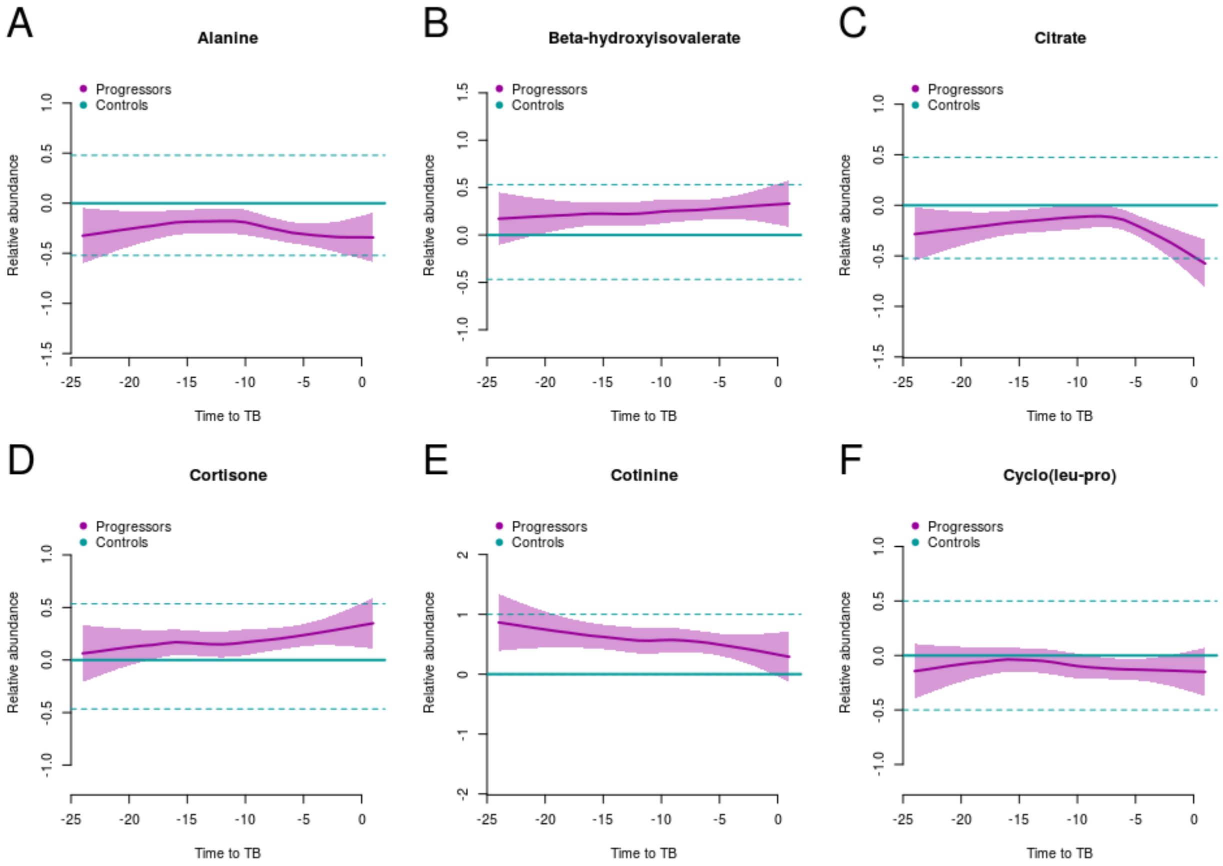

A. M.31453: cysteine; B. M.2730: gamma-glutamylglutamine; C. M.43829: gamma-glutamylvaline; D. M.53: glutamine; E. M.32599: glycocholenate sulfate; F. M.42574: glycohyocholate;

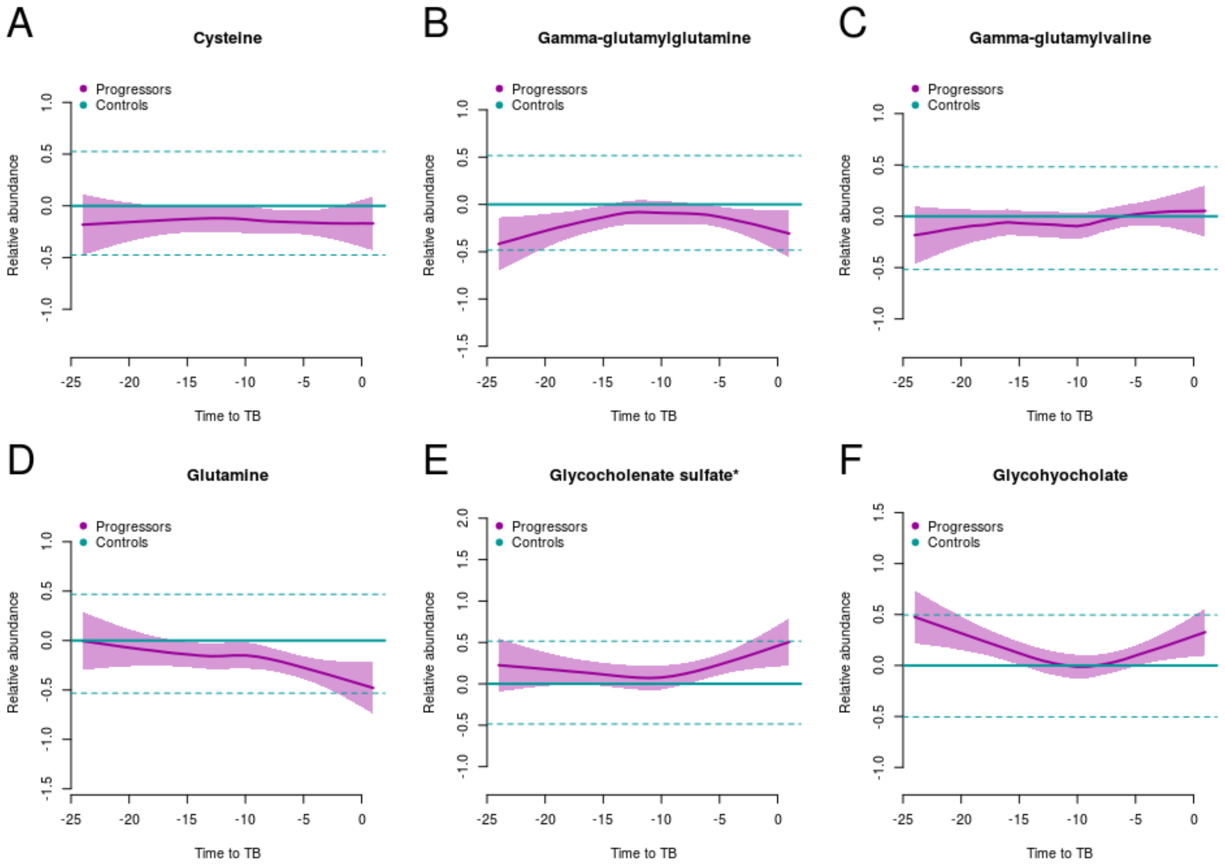

A. M.59: histidine; B. M.43264: 3-hydroxybutyrylcarnitine (1); C. M.46739: laurylcarnitine (C12); D. M.584: mannose; E. M.1302: methionine; F. M.33952: myristoylcarnitine (C14);

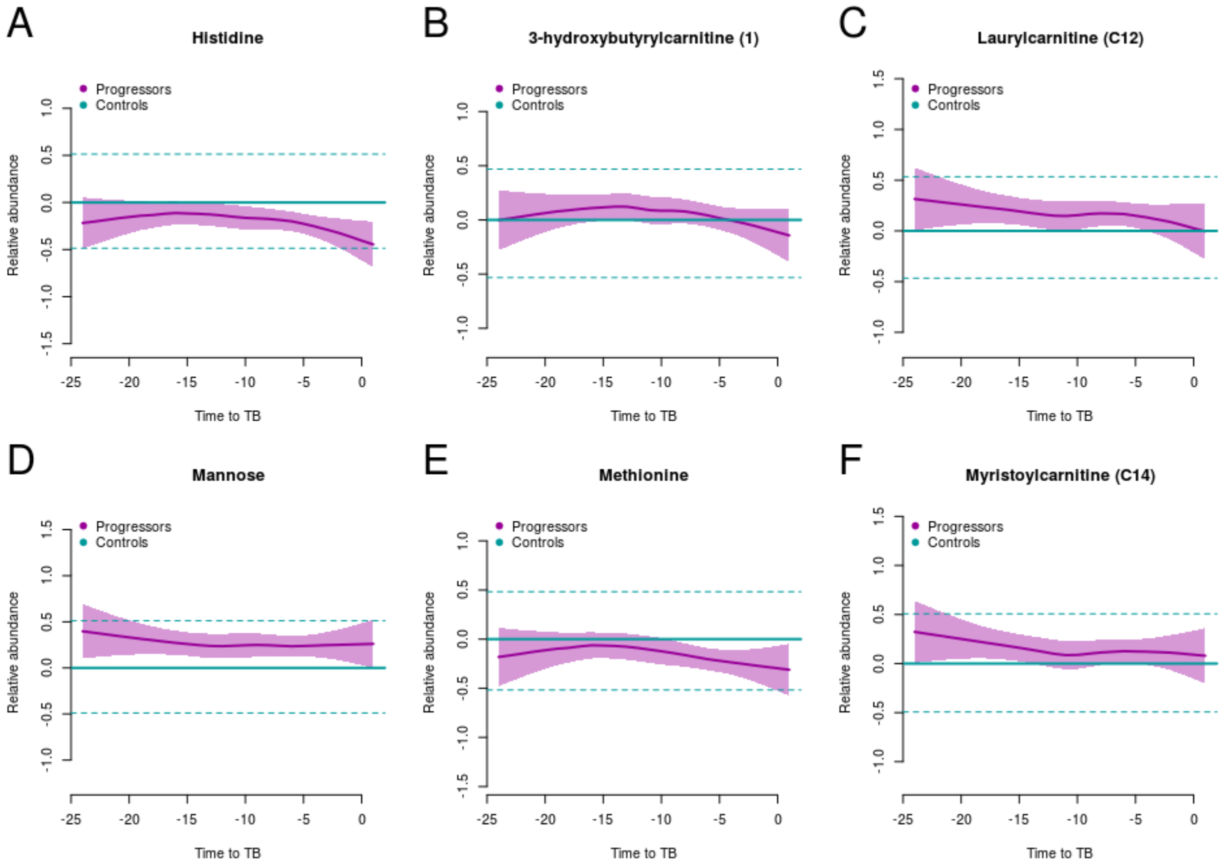

A. M.43488: N-acetylcarnosine; B. M.27710: N-acetylglycine; C. M.38102: oleoyl ethanolamide; D. M.35160: oleoylcarnitine (C18:1); E. M.1444: pipecolate; F. M.33935: piperine;

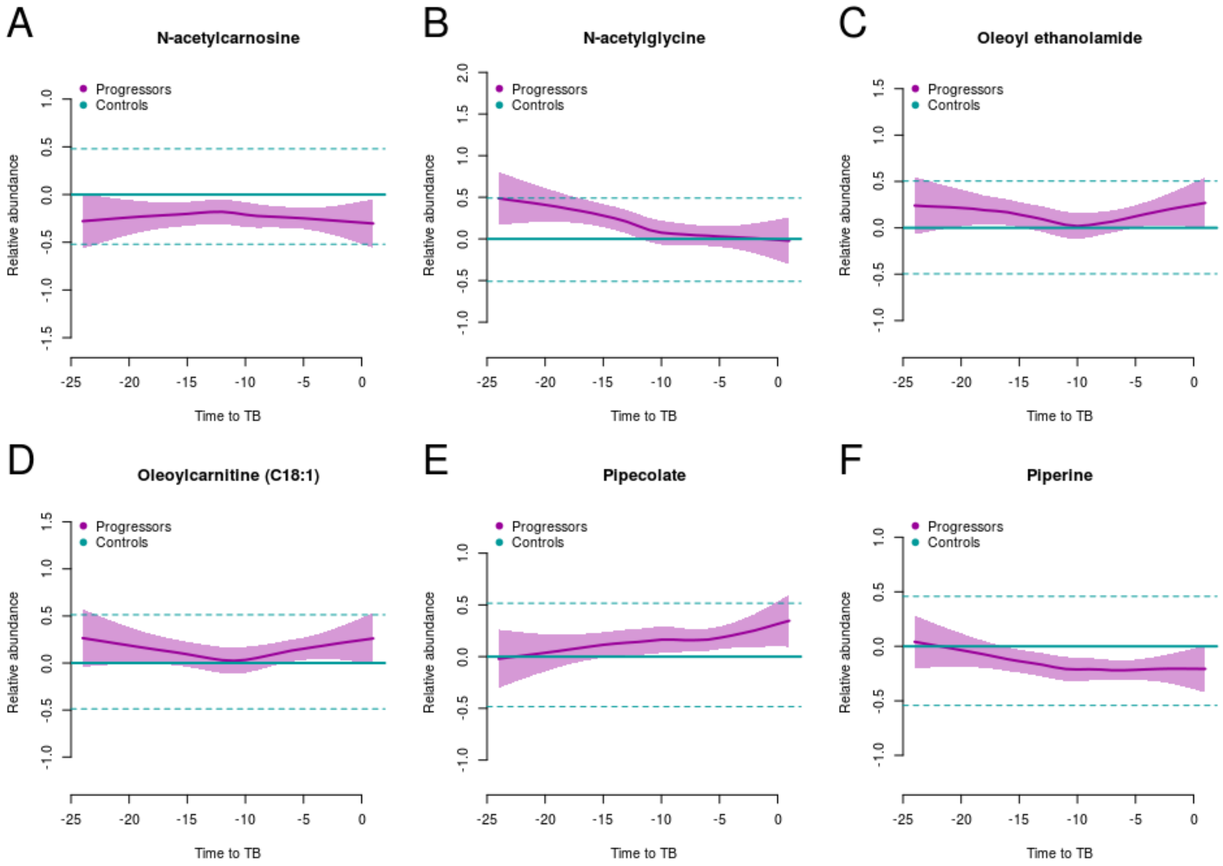

A. M.32562: pregnenediol disulfate (C21H34O8S2); B. M.15053: sorbitol; C. M.34445: sphingosine 1-phosphate; D. M.34409: stearoylcarnitine (C18); E. M.20693: tartronate (hydroxymalonate); F. M.27738: threonate;

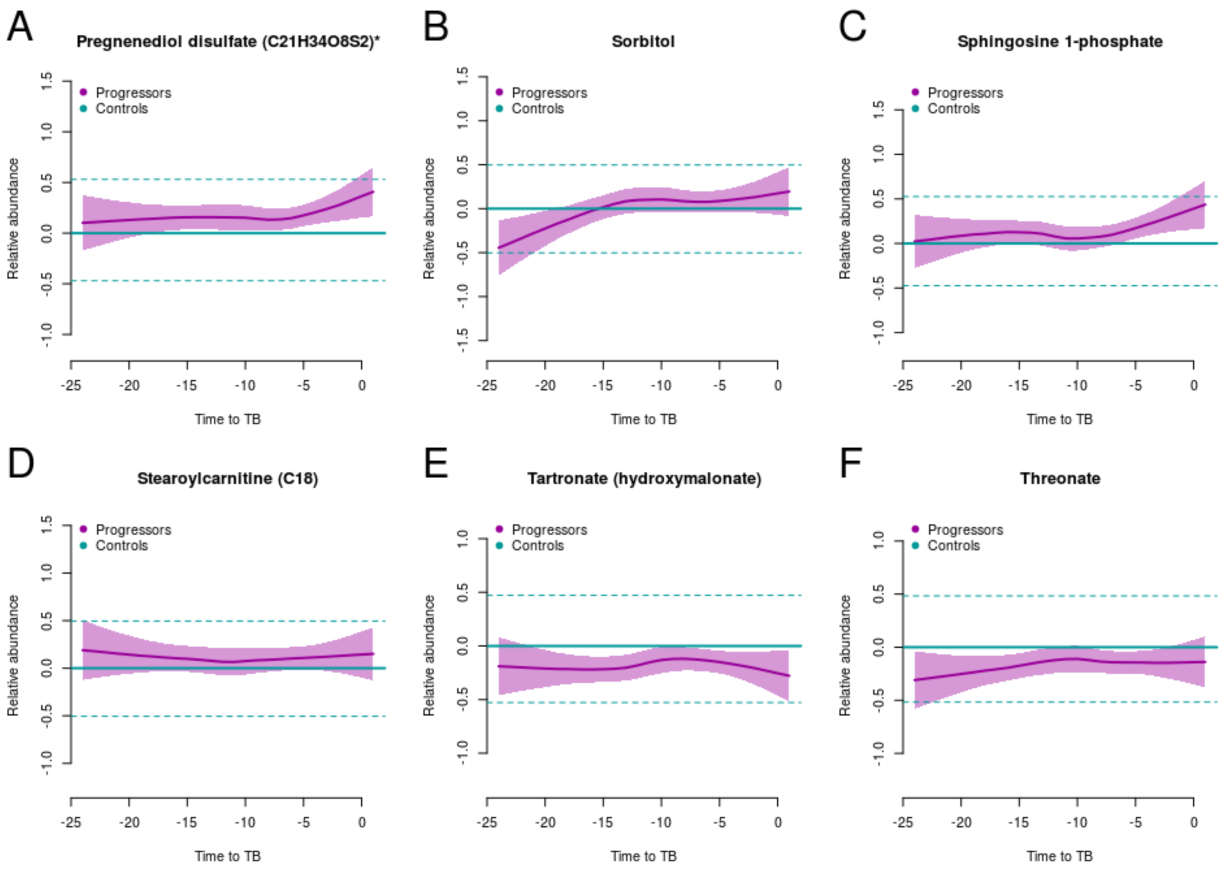

A. M.54: tryptophan; B. M.46595: X - 11378; C. M.33140: X - 11795; D. M.46608: X - 11880; E. M.47702: X - 12127; F. M.47709: 2-oxoarginine;

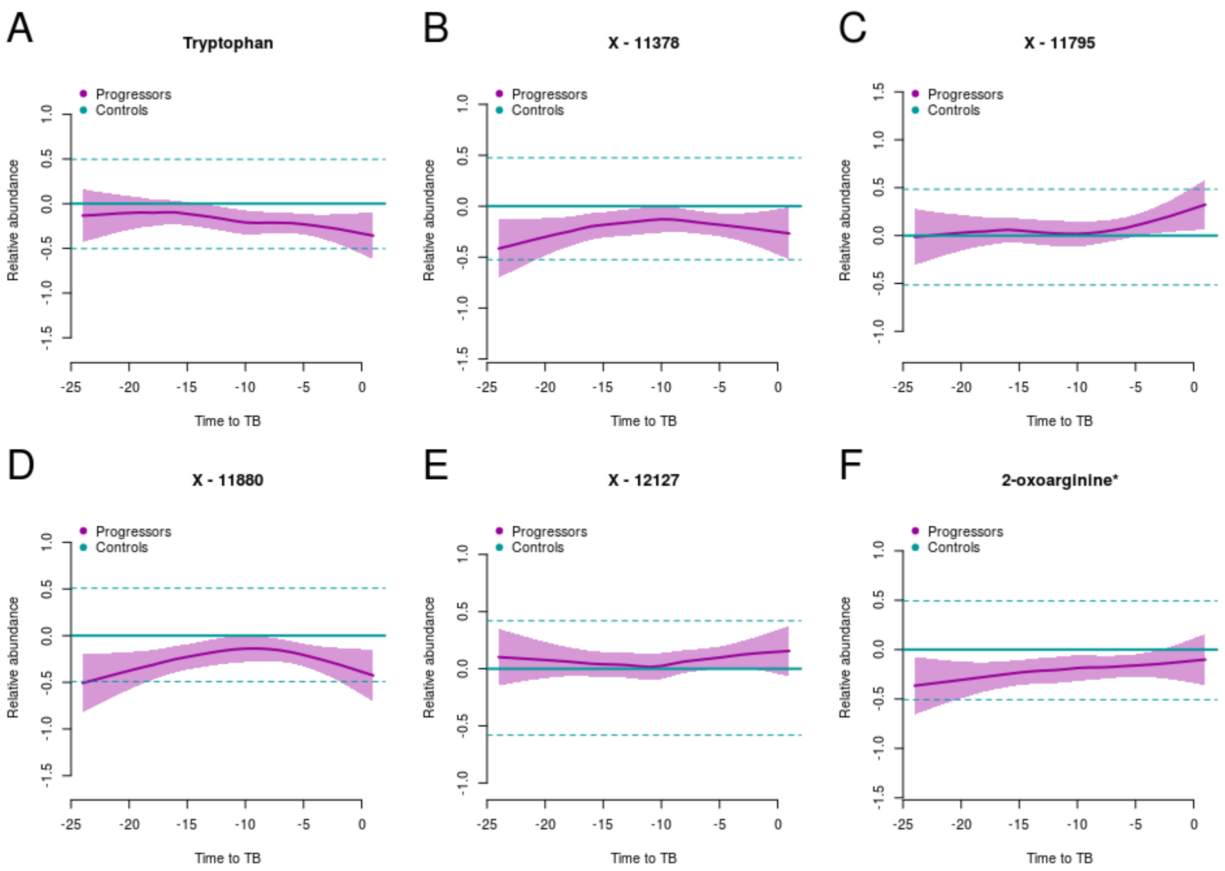

A. M.47715: argininate; B. M.46626: X - 12739; C. M.46622: thioproline; D. M.46471: hexanoylglutamine; E. M.46751: 3-hydroxybutyrylcarnitine (2); F. M.46652: X - 14658;

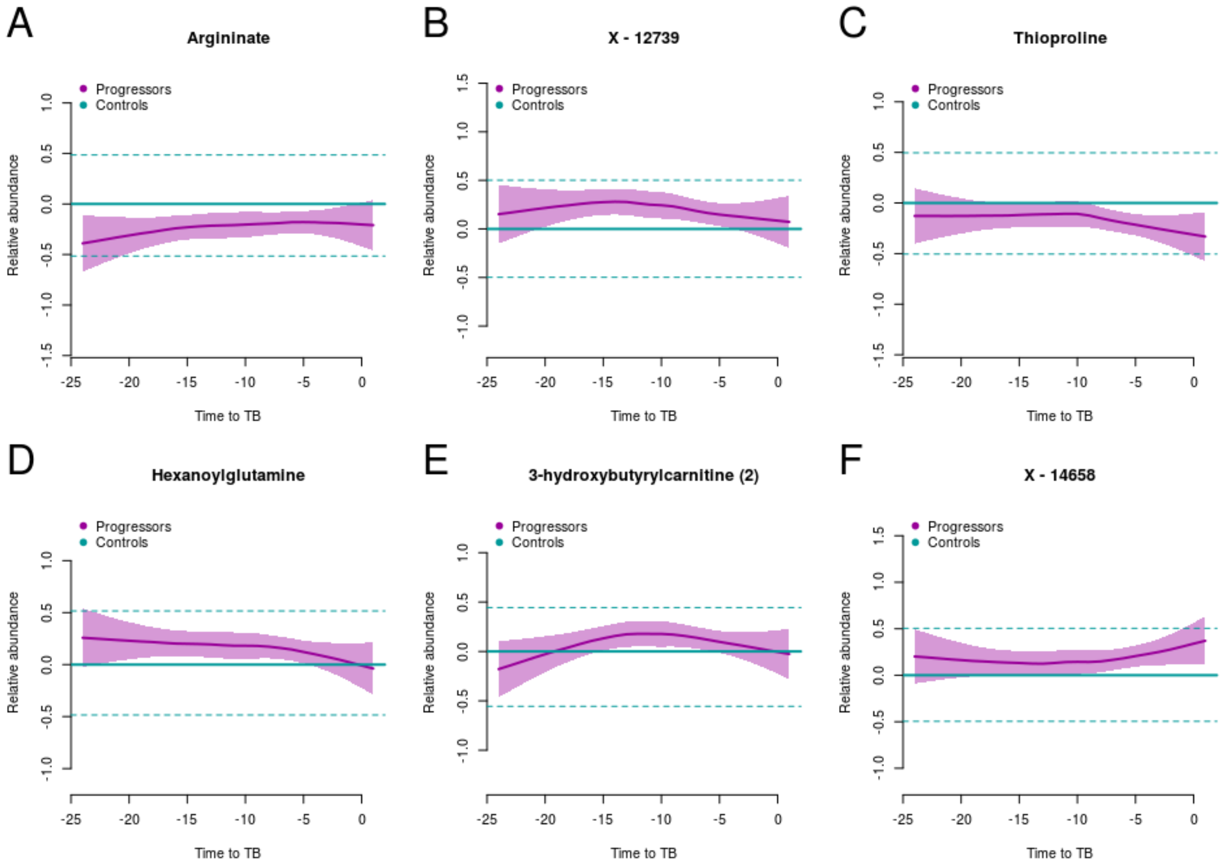

A. M.47986: eugenol sulfate; B. M.46366: X - 18249; C. M.46660: myristoleoylcarnitine; D. M.46368: X - 18914; E. M.46294: X - 21285; F. M.46515: X - 21470;

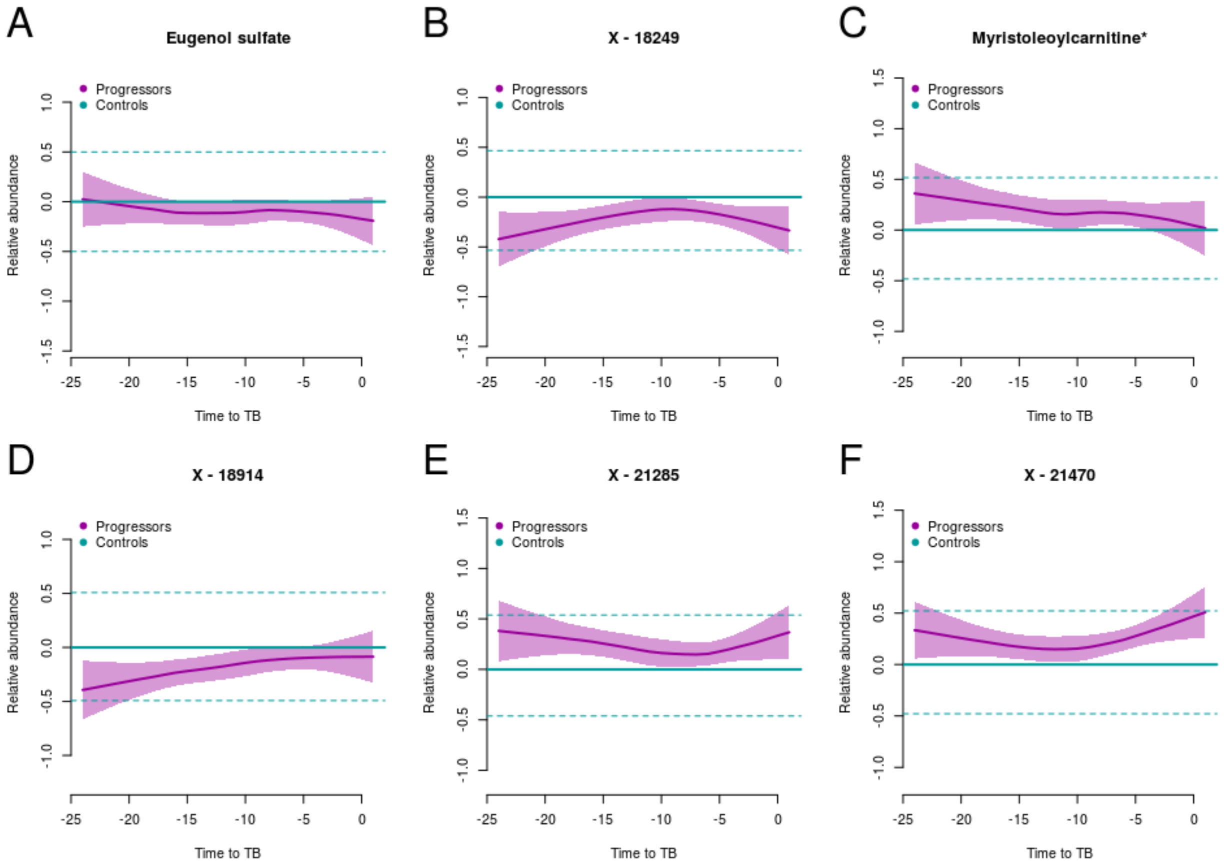

A. M.46905: X - 21736; B. M.46928: valine ion; C. M.46929: X - 21756; D. M.47391: 2'-O-methyluridine;

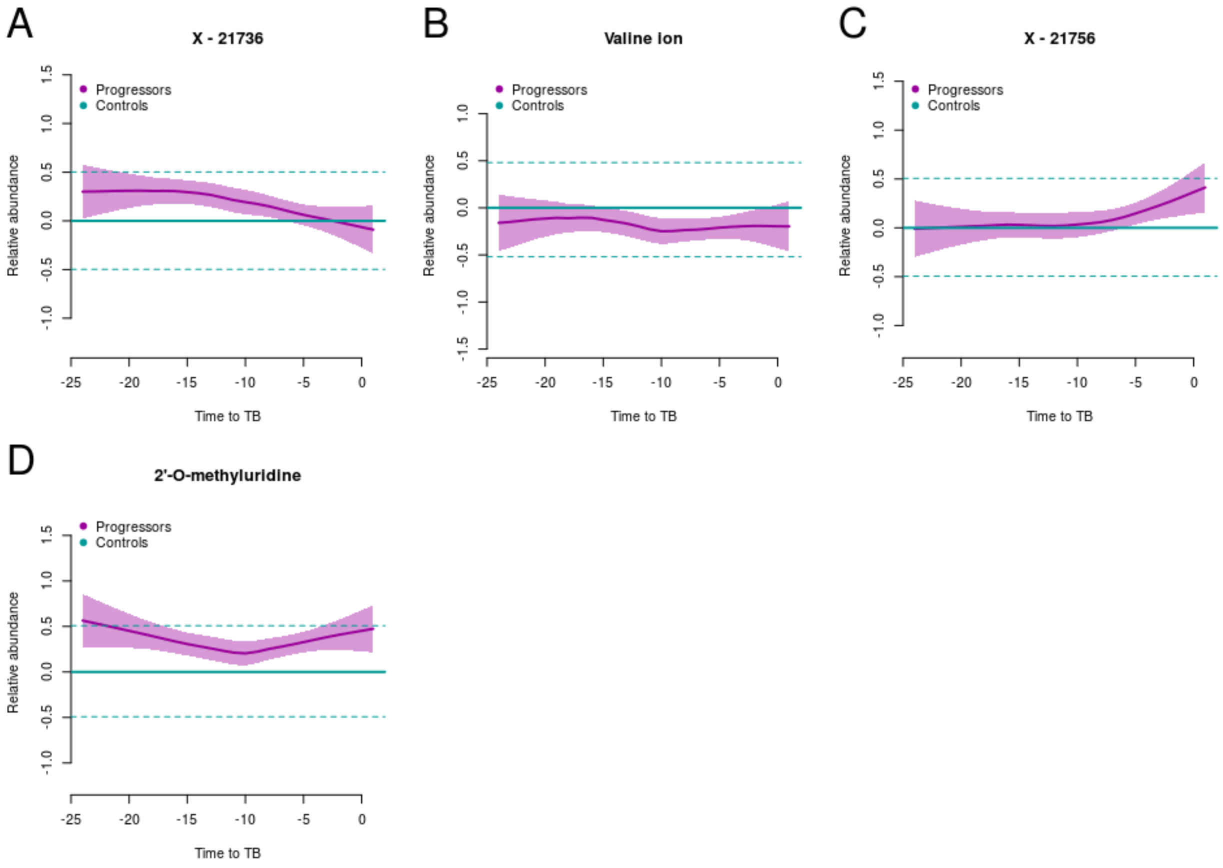

Supplementary Figure 8. *Smoking status and cotinine levels.* A, *number of smokers by study site. Grey, no data available; blue, smokers; mustard, non-smokers; green, individuals reporting to have quit smoking;* B, *cotinine levels and smoking status (> 6M, more than six months since quitting smoking; < 6M, less than six months since quitting);* C, *cotinine levels and smoking frequency (in cigarettes per day; N.a. – not applicable to non-smokers);* D, *cotinine levels and smoking duration (in years; N.a. – not applicable to non-smokers).*

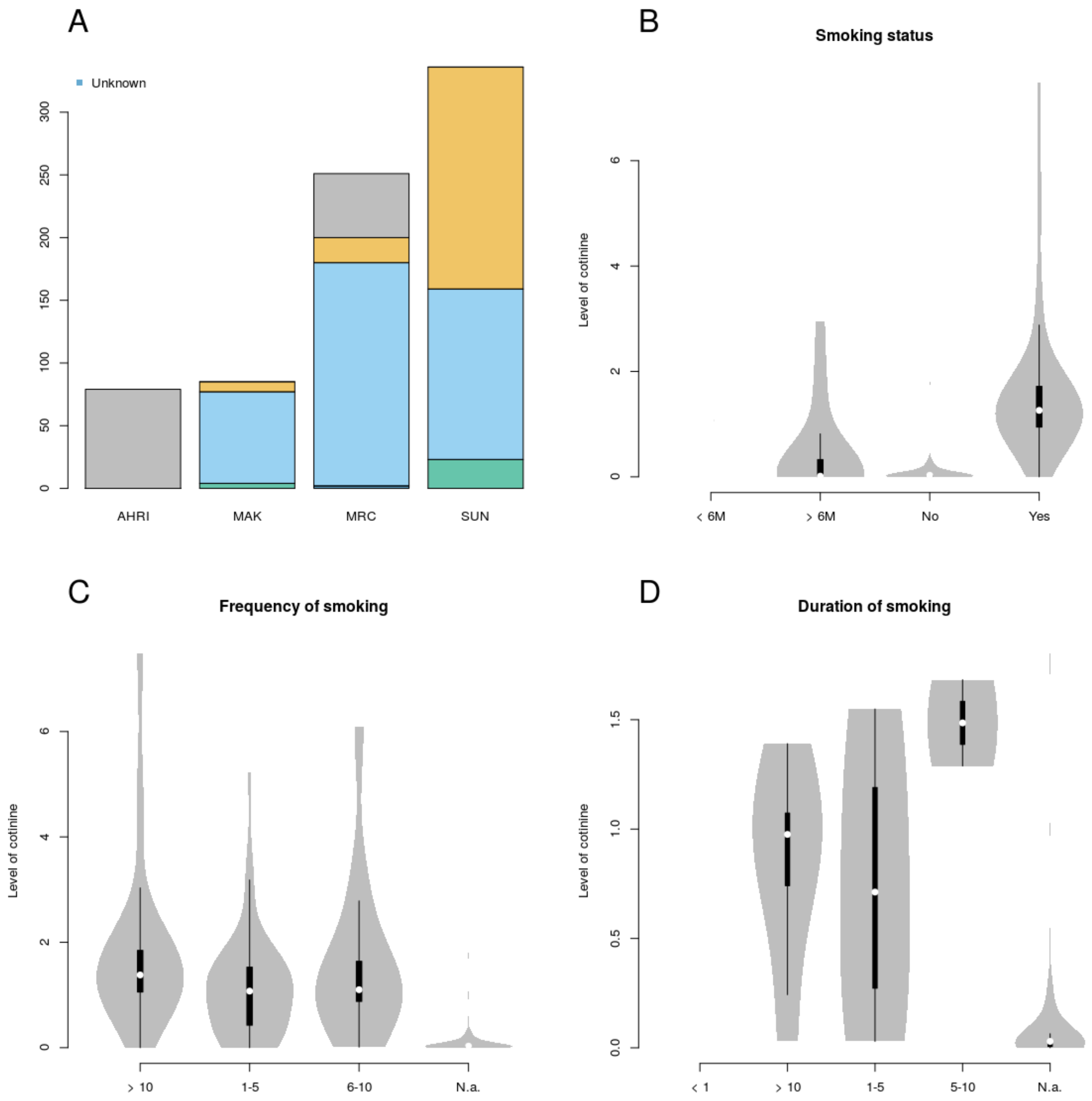

Supplementary Tables

Supplementary Table 1. GC6-74 recruitment and follow up dates.

Country	Institution	First date of enrolment	Last date of enrolment	Last date of follow-up	Number of Index cases	Number of Household Contacts
South Africa	SUN	February 27th, 2006	December 14th, 2010	November 28th, 2012	209	1,197
The Gambia	MRC	March 5th, 2007	October 21st, 2010	October 22nd, 2012	402	1,948
Uganda	MAK	June 1st, 2006	June 8th, 2010	May 4th, 2012	181	499
Ethiopia	AHRI	February 12th, 2007	August 3rd, 2011	August 16th, 2012	154	818

Supplementary Table 2. Criteria for tuberculosis diagnosis in GC6-74 progressors.

Category	Culture 1	Culture 2†	AFB 1	AFB 2†	CXR	Symptoms	TB Treatment Response	Failed AB	New Class
A	+	+							Definite
B	+				+				Definite
C	+		+						Definite
D			+	+	+				Probable
E			+	+		+			Probable
F			+		+				Probable
G	+					+	+		Probable
H	+					+			Possible
I			+	+			+		Possible
J			+			+			Possible
K					+	+	+	+	Possible
L	+								Questionable
M			+	+					Questionable
N			+						Questionable
O					+	+			Questionable
P					+				Questionable
Q						+			Questionab

								le
R	Neg/ND	Neg/ND	Neg/ND	Neg/ND	Neg/ND	+	Rx not started	Non-TB case
S	Neg/ND	Neg/ND	Neg/ND	Neg/ND	Neg/ND	Neg/ND	Rx not started	Non-TB case

Culture
Either liquid or solid agar positive with confirmed speciation for M.tuberculosis complex

AFB
Acid-fast bacilli: sputum smear ≥scanty (≥1-9 acid-fast organisms per 100x oil field)

CXR
Chest X-ray compatible with active TB

Failed AB
Failed antibiotics treatment; no response of symptoms to a 7-day, broad spectrum oral or IV antibiotics

†
Positivity on a separate sample of culture or AFB (not an aliquot of the same sample) provided by the participant preferrably but not necessarily on separate days

Note: Questionable classification is excluded in both progressors and non-progressors (controls)

Supplementary Table 3. Institutional Review Boards that provided ethics approvals for the different GC6-74 sites

Institution	Country	Ethics Review Committee	Protocol no.
SUN	South Africa	Stellenbosch University Institutional Review Board	N05/11/187
MRC	The Gambia	Joint Medical Research Council and Gambian Government	SCC.1141vs2
MAK	Uganda	Uganda National Council for Science and Technology	MV 715
MAK	Uganda	University Hospitals Case Medical Centre	12-95-08
AHRI	Ethiopia	Armauer Hansen Research Institute (AHRI)/All Africa Leprosy, TB and Rehabilitation Training Center (ALERT)	P015/10
UCT	South Africa	University of Cape Town Human Research Ethics Committee HREC	013/2013

Supplementary Table 4. Summary of the results of the tuberculin skin test (TST) at the baseline and study end. Numbers of persons with the result of TST greater than 10 mm are stratified by the GC-74 sites.

	Baseline	Any time point
**South Africa**	120 (85.7 %)	123 (87.9 %)
**The Gambia**	41 (36.3 %)	47 (41.6 %)
**Ethiopia**	18 (51.4 %)	18 (51.4 %)
**Uganda**	39 (100.0 %)	39 (100.0 %)

Supplementary Table 5. Sample types by cohort. *P*, progressors, *C*, controls.

	Plasma	Plasma/RPMI	Serum
**AHRI**	79 (P=20, C=59)		
**MAK**		45 (P=10, C=35)	40 (P=9, C=31)
**MRC**	251 (P=61, C=190)		
**SUN**	36 (P=11, C=25)		300 (P=70, C=230)

Supplementary Table 6. Performance of the different machine learning models in internal k-fold cross-validation within the training data sets. The models were trained and evaluated on the training data set. *Site*, the name of the site from which the samples were used; *Timepoints*, the selected timepoints, either all samples, or samples close (proximate) to the diagnoses, or samples further away (distal) from the diagnosis (see column 2). *AUC*, Area Under Curve; *CI*, 95% confidence interval; *p-value*, p-value associated with the AUC; *q-value*, p-value adjusted with the Benjamini-Hochberg procedure; *PPV*, positive predictive value; *NPV*, negative predictive value; *SENS*, sensitivity at 0.75 specificity; *PPV*^{2} and *NPV*^{2}: PPV and NPV assuming 2% prevalence and at 0.75 sensitivity; *N*, number of features used in the model.

Site	Timepoints	AUC	p-value	q-value	PPV	NPV	SENS	PPV^2	NPV^2	N
SUN	all	0.63 (0.55-0.71)	0.0032	**0.029**	0.31 (0.25-0.38)	0.97 (0.87-1.00)	0.36	0.029	0.98	866
	distal	0.65 (0.57-0.72)	0.0013	**0.013**	0.34 (0.25-0.44)	0.85 (0.78-0.91)	0.35	0.028	0.98	866
	proximate	0.76 (0.55-0.98)	0.018	0.12	0.17 (0.06-0.35)	0.99 (0.95-1.00)	0.71	0.055	0.99	866
MRC	all	0.71 (0.61-0.81)	5.5e-05	**0.00066**	0.68 (0.49-0.83)	0.85 (0.78-0.90)	0.57	0.045	0.99	866
	distal	0.61 (0.49-0.73)	0.061	0.37	0.32 (0.18-0.50)	0.86 (0.78-0.91)	0.42	0.033	0.98	866
	proximate	0.83 (0.70-0.95)	0.00012	**0.0013**	0.36 (0.18-0.57)	0.97 (0.91-0.99)	0.69	0.053	0.99	866
AHRI	all	0.57 (0.38-0.76)	0.46	1	0.39 (0.17-0.64)	0.82 (0.65-0.93)	0.46	0.036	0.99	866
	distal	0.57 (0.34-0.80)	0.53	1	0.33 (0.12-0.62)	0.88 (0.71-0.96)	0.5	0.039	0.99	866
	proximate	0.66 (0.27-1.00)	0.31	1	0.40 (0.05-0.85)	0.95 (0.82-0.99)	0.5	0.039	0.99	866
MAK	all	0.56 (0.40-0.73)	0.51	1	0.32 (0.17-0.51)	0.91 (0.71-0.99)	0.17	0.013	0.98	682
	distal	0.55 (0.39-0.71)	0.61	1	0.30 (0.16-0.49)	0.95 (0.74-1.00)	0.091	0.0074	0.98	682
TOT	all	0.64 (0.59-0.70)	1.6e-06	**2.2e-05**	0.45 (0.35-0.56)	0.79 (0.75-0.83)	0.39	0.031	0.98	644
	distal	0.59 (0.53-0.65)	0.0063	0.05	0.26 (0.21-0.31)	0.86 (0.80-0.90)	0.32	0.025	0.98	644
	proximate	0.77 (0.67-0.87)	6.8e-06	**8.8e-05**	0.27 (0.15-0.42)	0.97 (0.94-0.98)	0.63	0.049	0.99	644

Supplementary Table 7. Performance of the machine learning tested cross-wise between cohorts within the training set. The models were trained and evaluated only on the training data set. *Train*, the name of the site from which the samples were used for testing; *Test*, name of the site on which the given model was tested. *AUC*, Area Under Curve; *CI*, 95% confidence interval; *p-value*, p-value associated with the AUC; *q-value*, p-value adjusted with the Benjamini-Hochberg procedure; *PPV*, positive predictive value; *NPV*, negative predictive value; *SENS*, sensitivity at 0.75 specificity; *PPV²* and *NPV²*: PPV and NPV assuming 2% prevalence and at 0.75 sensitivity; *N*, number of features in a model-test set combination.

Train	Test	AUC	p-value	q-value	PPV	NPV	SENS	PPV²	NPV²	N
SUN	MRC	0.70 (0.61-0.79)	0.00012	**0.0014**	0.46 (0.31-0.61)	0.83 (0.76-0.90)	0.54	0.043	0.99	866
	AHRI	0.57 (0.40-0.75)	0.44	1	0.12 (0.02-0.38)	0.69 (0.51-0.83)	0.38	0.03	0.98	866
	MAK	0.60 (0.42-0.77)	0.32	1	0.29 (0.15-0.46)	0.93 (0.68-1.00)	0.33	0.026	0.98	644
MRC	SUN	0.64 (0.56-0.73)	0.00084	**0.0093**	0.40 (0.29-0.52)	0.81 (0.73-0.86)	0.46	0.036	0.99	866
	AHRI	0.56 (0.38-0.75)	0.51	1	0.18 (0.06-0.37)	0.65 (0.43-0.84)	0.23	0.018	0.98	866
	MAK	0.55 (0.35-0.75)	0.62	1	0.40 (0.12-0.74)	0.81 (0.67-0.92)	0.33	0.026	0.98	644
AHRI	SUN	0.52 (0.43-0.60)	0.69	1	0.24 (0.17-0.31)	0.68 (0.55-0.79)	0.34	0.027	0.98	866
	MRC	0.56 (0.46-0.65)	0.26	1	0.31 (0.22-0.41)	0.83 (0.73-0.91)	0.26	0.021	0.98	866
	MAK	0.66 (0.50-0.82)	0.097	0.88	0.35 (0.19-0.55)	0.95 (0.77-1.00)	0.33	0.026	0.98	644
MAK	SUN	0.60 (0.52-0.69)	0.019	0.19	0.37 (0.27-0.47)	0.82 (0.74-0.88)	0.39	0.031	0.98	644
	MRC	0.53 (0.43-0.64)	0.5	1	0.35 (0.20-0.53)	0.78 (0.70-0.85)	0.34	0.027	0.98	644
	AHRI	0.61 (0.43-0.79)	0.25	1	0.34 (0.19-0.53)	0.89 (0.67-0.99)	0.23	0.018	0.98	644

Supplementary Table 8. Performance of the machine learning tested cross-wise between sample types within the training set. The models were trained and evaluated only on the training data set. *Train*, sample type from which the samples were used for testing; *Test*, name of the site on which the given model was tested. *AUC*, Area Under Curve; *CI*, 95% confidence interval; *p-value*, p-value associated with the AUC; *q-value*, p-value adjusted with the Benjamini-Hochberg procedure; *PPV*, positive predictive value; *NPV*, negative predictive value; *SENS*, sensitivity at 0.75 specificity; PPV^2 and NPV^2: PPV and NPV assuming 2% prevalence and at 0.75 sensitivity; *N*, number of features in a model-test set combination.

Train	Subset	Test	AUC	p-value	q-value	PPV	NPV	SENS	PPV^2	NPV^2	N
plasma	all	serum	0.66 (0.58-0.75)	0.00022	**0.00044**	0.46 (0.31-0.61)	0.81 (0.74-0.86)	0.41	0.032	0.98	866
		rpmi	0.67 (0.45-0.90)	0.21	0.21	0.35 (0.14-0.62)	1.00 (0.69-1.00)	0.5	0.039	0.99	683
	distal	serum	0.64 (0.55-0.72)	0.0023	**0.0046**	0.50 (0.34-0.66)	0.81 (0.74-0.86)	0.46	0.037	0.99	866
		rpmi	0.65 (0.38-0.92)	0.29	0.29	0.44 (0.14-0.79)	0.89 (0.65-0.99)	0.67	0.052	0.99	683
	proximate	serum	0.60 (0.52-0.69)	0.019	**0.037**	0.50 (0.31-0.69)	0.79 (0.73-0.85)	0.38	0.03	0.98	866
		rpmi	0.48 (0.25-0.71)	0.91	0.91	0.00 (0.00-0.52)	0.73 (0.50-0.89)	0	0	0.97	683
serum	all	plasma	0.66 (0.59-0.74)	8e-05	**0.00016**	0.39 (0.30-0.50)	0.82 (0.76-0.88)	0.44	0.034	0.98	866
		rpmi	0.76 (0.52-1.00)	0.058	0.058	0.50 (0.16-0.84)	0.89 (0.67-0.99)	0.67	0.052	0.99	682
	distal	plasma	0.65 (0.57-0.73)	0.00028	**0.00057**	0.38 (0.29-0.47)	0.85 (0.78-0.91)	0.43	0.034	0.98	866
		rpmi	0.73 (0.44-1.00)	0.1	0.1	0.44 (0.14-0.79)	0.89 (0.65-0.99)	0.67	0.052	0.99	682
	proximate	plasma	0.63 (0.54-0.71)	0.0024	**0.0048**	0.42 (0.31-0.53)	0.81 (0.75-0.87)	0.51	0.04	0.99	866
		rpmi	0.57 (0.26-0.88)	0.64	0.64	0.67 (0.09-0.99)	0.83 (0.63-0.95)	0.33	0.026	0.98	682
rpmi	all	plasma	0.56 (0.47-0.64)	0.18	0.18	0.32 (0.23-0.43)	0.78 (0.70-0.84)	0.29	0.023	0.98	683
		serum	0.62 (0.53-0.71)	0.0081	**0.016**	0.56 (0.38-0.74)	0.81 (0.74-0.86)	0.41	0.032	0.98	682
	distal	plasma	0.57 (0.48-0.66)	0.087	0.087	0.37 (0.26-0.49)	0.78 (0.72-0.84)	0.41	0.032	0.98	683
		serum	0.59 (0.50-0.68)	0.04	0.081	0.38 (0.26-0.52)	0.80 (0.73-0.86)	0.41	0.032	0.98	682

Supplementary Table 9. The top 25 compounds (by mean decrease in accuracy) in the models Total and Total/BL. *Mean decrease*: mean decrease in accuracy.

	Model	ID	Name	Mean decrease
**M.32599**	Total	M.32599	glycocholenate sulfate*	0.01
**M.64**		M.64	phenylalanine	0.0081
**M.31453**		M.31453	cysteine	0.0061
**M.59**		M.59	histidine	0.0058
**M.2132**		M.2132	citrulline	0.0055
**M.584**		M.584	mannose	0.0043
**M.1712**		M.1712	cortisol	0.004
**M.53**		M.53	glutamine	0.0037
**M.54**		M.54	tryptophan	0.0034
**M.2730**		M.2730	gamma-glutamylglutamine	0.0033
**M.31787**		M.31787	3-carboxy-4-methyl-5-propyl-2-furanpropanoate (CMPF)	0.0028
**M.32807**		M.32807	taurocholenate sulfate	0.0027
**M.12129**		M.12129	beta-hydroxyisovalerate	0.0027
**M.15140**		M.15140	kynurenine	0.0021
**M.1564**		M.1564	citrate	0.002
**M.3127**		M.3127	hypoxanthine	0.0018
**M.27718**		M.27718	creatine	0.0014
**M.37202**		M.37202	androstenediol (3beta,17beta) disulfate (1)	0.0014
**M.32836**		M.32836	HWESASXX*	0.0013
**M.37231**		M.37231	1-docosapentaenoyl-GPC (22:5n3)*	0.0013
**M.33822**		M.33822	1-docosahexaenoyl-GPC (22:6)*	0.0012
**M.34456**		M.34456	gamma-glutamylisoleucine*	0.0012
**M.33937**		M.33937	alpha-hydroxyisovalerate	0.0012

**M.15122**		M.15122	glycerol	0.0012
**M.3141**		M.3141	betaine	0.0011
**M.5841**	Total/BL	M.584	mannose	0.0063
**M.314531**		M.31453	cysteine	0.0061
**M.325991**		M.32599	glycocholenate sulfate*	0.0044
**M.641**		M.64	phenylalanine	0.0043
**M.121291**		M.12129	beta-hydroxyisovalerate	0.0031
**M.317871**		M.31787	3-carboxy-4-methyl-5-propyl-2-furanpropanoate (CMPF)	0.0031
**M.542**		M.542	3-hydroxybutyrate (BHBA)	0.0027
**M.17121**		M.1712	cortisol	0.0023
**M.328071**		M.32807	taurocholenate sulfate	0.0019
**M.21321**		M.2132	citrulline	0.0017
**M.27301**		M.2730	gamma-glutamylglutamine	0.0017
**M.277181**		M.27718	creatine	0.0017
**M.32980**		M.32980	adrenate (22:4n6)	0.0015
**M.35159**		M.35159	cysteine-glutathione disulfide	0.0014
**M.591**		M.59	histidine	0.0014
**M.18349**		M.18349	indolelactate	0.0013
**M.3147**		M.3147	xanthine	0.0013
**M.531**		M.53	glutamine	0.0012
**M.32786**		M.32786	hydroxy-CMPF*	0.0012
**M.33941**		M.33941	decanoylcarnitine (C10)	0.0012
**M.27710**		M.27710	N-acetylglycine	0.0012
**M.15641**		M.1564	citrate	0.001
**M.18467**		M.18467	eicosapentaenoate (EPA; 20:5n3)	0.00099
**M.32857**		M.32857	5-dodecenoylcarnitine	0.00092
**M.541**		M.54	tryptophan	0.00092

Supplementary Table 10. Performance of the different machine learning models on the blinded data sets. All ML models were trained on the training data set. *Model*, the name of the model applied either to all samples, or samples close (proximate) to the diagnoses, or samples further away (distal) from the diagnosis (see column 2). *AUC*, Area Under Curve; *CI*, 95% confidence interval; *p-value*, p-value associated with the AUC; *q-value*, p-value adjusted with the Benjamini-Hochberg procedure; *PPV*, positive predictive value; *NPV*, negative predictive value; *SENS*, sensitivity at 0.75 specificity; PPV^2 and NPV^2: PPV and NPV assuming 2% prevalence and at 0.75 sensitivity; *N*, number of features.

Model	Timepoints	AUC	p-value	q-value	PPV	NPV	SENS	PPV^2	NPV^2	N
Distal serum	all	0.61 (0.52-0.71)	0.011	0.21	0.32 (0.23-0.41)	0.86 (0.79-0.92)	0.47	0.037	0.99	571
	proximate	0.68 (0.53-0.83)	0.029	0.44	0.35 (0.20-0.53)	0.91 (0.76-0.98)	0.44	0.034	0.98	
	distal	0.59 (0.48-0.70)	0.1	0.96	0.30 (0.20-0.42)	0.85 (0.76-0.91)	0.46	0.036	0.99	
	BL	0.56 (0.45-0.68)	0.27	1	0.30 (0.20-0.43)	0.85 (0.76-0.92)	0.32	0.025	0.98	
BL serum	all	0.59 (0.50-0.68)	0.045	0.54	0.36 (0.23-0.50)	0.82 (0.76-0.88)	0.38	0.03	0.98	571
	proximate	0.58 (0.41-0.75)	0.32	1	0.30 (0.17-0.46)	0.89 (0.72-0.98)	0.38	0.03	0.98	
	distal	0.59 (0.48-0.70)	0.096	0.96	0.40 (0.24-0.58)	0.83 (0.76-0.89)	0.43	0.034	0.98	
	BL	0.58 (0.46-0.69)	0.19	1	0.35 (0.20-0.54)	0.82 (0.75-0.89)	0.36	0.029	0.98	
Distal plasma	all	0.60 (0.51-0.70)	0.021	0.36	0.36 (0.24-0.50)	0.83 (0.77-0.88)	0.42	0.033	0.98	571
	proximate	0.69 (0.53-0.85)	0.022	0.36	0.57 (0.29-0.82)	0.86 (0.74-0.94)	0.5	0.039	0.99	
	distal	0.57 (0.46-0.68)	0.22	1	0.42 (0.22-0.63)	0.82 (0.75-0.88)	0.38	0.03	0.98	
	BL	0.54 (0.42-0.65)	0.52	1	0.25 (0.16-0.36)	0.83 (0.72-0.91)	0.33	0.026	0.98	
BL plasma	all	0.64 (0.55-0.72)	0.0026	0.061	0.45 (0.30-0.60)	0.84 (0.78-0.89)	0.45	0.036	0.99	571
	proximate	0.45 (0.27-0.62)	0.51	1	0.20 (0.09-0.36)	0.74 (0.55-0.88)	0.12	0.01	0.98	
	distal	0.68 (0.58-0.77)	0.0011	**0.027**	0.31 (0.22-0.42)	0.88 (0.79-0.94)	0.49	0.038	0.99	
	BL	0.65 (0.54-0.75)	0.011	0.21	0.41 (0.25-0.59)	0.84 (0.76-0.90)	0.45	0.036	0.99	
TotalF	all	0.71 (0.62-0.79)	4.2e-06	**0.00013**	0.81 (0.61-0.93)	0.85 (0.80-0.90)	0.53	0.041	0.99	192
	proximate	0.78 (0.62-0.94)	0.00071	**0.019**	1.00 (0.66-1.00)	0.89 (0.78-0.95)	0.69	0.053	0.99	
	distal	0.68 (0.58-0.79)	0.00071	**0.019**	0.80 (0.52-0.96)	0.84 (0.78-0.89)	0.46	0.036	0.99	
	BL	0.64 (0.52-0.76)	0.017	0.3	0.71 (0.42-0.92)	0.84 (0.76-0.89)	0.42	0.033	0.98	
Total BL	all	0.73 (0.65-0.81)	2.7e-07	**8.7e-06**	0.59 (0.42-0.74)	0.85 (0.80-0.90)	0.57	0.044	0.99	192
	proximate	0.75 (0.59-0.91)	0.0026	0.061	0.64 (0.35-0.87)	0.88 (0.76-0.95)	0.62	0.049	0.99	
	distal	0.72 (0.63-0.82)	3.2e-05	**0.00097**	0.33 (0.23-0.44)	0.89 (0.81-0.95)	0.54	0.042	0.99	
	BL	0.71 (0.61-0.81)	0.00021	**0.006**	0.34 (0.23-0.47)	0.89 (0.81-0.95)	0.55	0.043	0.99	

GGC	all	0.59 (0.50-0.67)	0.041	0.54	0.27 (0.20-0.36)	0.86 (0.78-0.92)	0.38	0.03	0.98	2
	proximate	0.59 (0.44-0.74)	0.28	1	0.31 (0.17-0.48)	0.88 (0.71-0.96)	0.31	0.025	0.98	
	distal	0.59 (0.49-0.70)	0.078	0.86	0.32 (0.19-0.47)	0.83 (0.75-0.89)	0.41	0.032	0.98	
	BL	0.53 (0.43-0.64)	0.54	1	0.26 (0.17-0.37)	0.84 (0.74-0.92)	0.24	0.019	0.98	
Compact	all	0.66 (0.58-0.75)	0.00026	**0.0072**	0.37 (0.27-0.47)	0.88 (0.82-0.93)	0.49	0.039	0.99	25
	proximate	0.72 (0.58-0.85)	0.0091	0.2	0.44 (0.25-0.65)	0.91 (0.78-0.97)	0.62	0.049	0.99	
	distal	0.64 (0.53-0.75)	0.0095	0.2	0.35 (0.24-0.47)	0.88 (0.80-0.93)	0.43	0.034	0.98	
	BL	0.62 (0.51-0.73)	0.03	0.44	0.34 (0.23-0.48)	0.87 (0.79-0.93)	0.36	0.029	0.98	

Supplementary Table 11. Performance of the machine learning models "Total" and "Total.BL" on the blinded data sets, stratified by site. All ML models were trained on the training data set. *Model*, the name of the model applied all samples from one of the four cohorts; *Site*, the subset of samples on which the model was evaluated. *AUC*, Area Under Curve; *CI*, 95% confidence interval; *p-value*, p-value associated with the AUC; *q-value*, p-value adjusted with the Benjamini-Hochberg procedure; *PPV*, positive predictive value; *NPV*, negative predictive value; *SENS*, sensitivity at 0.75 specificity; *PPV*^{2} and *NPV*^{2}: PPV and NPV assuming 2% prevalence and at 0.75 sensitivity. The number of features is given in Supplementary Table 10.

Model	Site	AUC	p-value	q-value	PPV	NPV	SENS	PPV^2	NPV^2
TotalF	SUN	0.80 (0.69-0.91)	4e-05	**0.00032**	0.41 (0.25-0.58)	0.92 (0.83-0.97)	0.65	0.05	0.99
	MRC	0.68 (0.51-0.84)	0.02	0.099	1.00 (0.63-1.00)	0.85 (0.75-0.92)	0.51	0.04	0.99
	AHRI	0.59 (0.28-0.91)	0.5	0.63	1.00 (0.29-1.00)	0.84 (0.64-0.95)	0.43	0.034	0.98
	MAK	0.63 (0.33-0.93)	0.32	0.63	1.00 (0.29-1.00)	0.86 (0.68-0.96)	0.43	0.034	0.98
Total BL	SUN	0.75 (0.62-0.88)	0.00053	**0.0037**	0.75 (0.43-0.95)	0.88 (0.79-0.94)	0.55	0.043	0.99
	MRC	0.73 (0.59-0.87)	0.0024	**0.014**	0.73 (0.39-0.94)	0.85 (0.74-0.92)	0.58	0.045	0.99
	AHRI	0.67 (0.39-0.95)	0.2	0.61	0.50 (0.16-0.84)	0.85 (0.62-0.97)	0.57	0.045	0.99
	MAK	0.72 (0.50-0.94)	0.087	0.35	0.43 (0.18-0.71)	0.94 (0.73-1.00)	0.57	0.045	0.99

Supplementary Table 12. Performance of the TB-HEALTHY model with 206 features when applied to GC6 data. *AUC*, area under curve; in parentheses, 95% confidence intervals; p-value for the AUC; q-value: p-value corrected using the Benjamini-Hochberg method; *PPV*, positive predictive value; *NPV*, negative predictive value; *SENS*, sensitivity at 0.75 specificity; *PPV*^{2} and *NPV*^{2}: PPV and NPV assuming 2% prevalence and at 0.75 sensitivity.

Site	Model	AUC	p-value	q-value	PPV	NPV	SENS	PPV^2	NPV^2
SUN	all	0.71 (0.64-0.78)	2e-08	**2.5e-07**	0.42 (0.33-0.51)	0.85 (0.80-0.90)	0.57	0.044	0.99
	distal	0.71 (0.64-0.78)	5.9e-08	**6.4e-07**	0.49 (0.37-0.62)	0.86 (0.81-0.90)	0.58	0.045	0.99
	proximate	0.81 (0.69-0.93)	0.00098	**0.0059**	0.09 (0.04-0.16)	0.99 (0.97-1.00)	0.6	0.047	0.99
MRC	all	0.74 (0.66-0.81)	3e-08	**3.6e-07**	0.58 (0.44-0.72)	0.85 (0.79-0.90)	0.56	0.044	0.99
	distal	0.68 (0.58-0.77)	3e-04	**0.0024**	0.47 (0.30-0.65)	0.87 (0.81-0.91)	0.5	0.039	0.99
	proximate	0.86 (0.75-0.96)	3.1e-07	**2.8e-06**	0.42 (0.26-0.59)	0.98 (0.94-0.99)	0.79	0.061	0.99
AHRI	all	0.55 (0.39-0.72)	0.48	1	1.00 (0.54-1.00)	0.81 (0.70-0.89)	0.34	0.027	0.98
	distal	0.45 (0.25-0.64)	0.54	1	0.50 (0.07-0.93)	0.84 (0.73-0.92)	0.15	0.012	0.98
	proximate	0.89 (0.75-1.00)	0.00077	**0.0054**	1.00 (0.48-1.00)	0.97 (0.89-1.00)	0.71	0.055	0.99
MAK	all	0.57 (0.43-0.72)	0.33	1	0.29 (0.17-0.44)	0.86 (0.71-0.95)	0.26	0.021	0.98
	distal	0.50 (0.34-0.67)	0.96	1	0.21 (0.11-0.34)	0.91 (0.72-0.99)	0.14	0.012	0.98
	proximate	0.75 (0.54-0.97)	0.061	0.3	0.14 (0.04-0.33)	0.98 (0.88-1.00)	0.6	0.047	0.99
TOT	all	0.68 (0.64-0.73)	1.1e-13	**1.6e-12**	0.35 (0.30-0.40)	0.86 (0.82-0.90)	0.5	0.039	0.99
	distal	0.65 (0.59-0.70)	8e-08	**8e-07**	0.30 (0.25-0.37)	0.86 (0.82-0.89)	0.47	0.037	0.99
	proximate	0.82 (0.75-0.89)	7.7e-12	**1.1e-10**	0.24 (0.16-0.33)	0.97 (0.95-0.98)	0.73	0.056	0.99

Supplementary Table 13. Performance of the shrinked TB-HEALTHY model with 10 features when applied to GC6 data. *AUC*, area under curve; in parentheses, 95% confidence intervals; p-value for the AUC; q-value: p-value corrected using the Benjamini-Hochberg method; *PPV*, positive predictive value; *NPV*, negative predictive value; *SENS*, sensitivity at 0.75 specificity; PPV^2 and NPV^2: PPV and NPV assuming 2% prevalence and at 0.75 sensitivity.

Site	Model	AUC	p-value	q-value	PPV	NPV	SENS	PPV^2	NPV^2
SUN	all	0.71 (0.64-0.78)	2.1e-08	**3e-07**	0.53 (0.41-0.65)	0.84 (0.79-0.88)	0.59	0.046	0.99
	distal	0.70 (0.62-0.77)	4.5e-07	**5e-06**	0.50 (0.38-0.62)	0.86 (0.81-0.90)	0.59	0.046	0.99
	proximate	0.78 (0.64-0.93)	0.0024	**0.017**	0.08 (0.04-0.14)	0.99 (0.96-1.00)	0.6	0.047	0.99
MRC	all	0.68 (0.60-0.76)	1.7e-05	**0.00017**	0.42 (0.31-0.54)	0.83 (0.77-0.89)	0.52	0.041	0.99
	distal	0.66 (0.56-0.75)	0.0015	**0.012**	0.34 (0.22-0.48)	0.87 (0.81-0.92)	0.48	0.037	0.99
	proximate	0.74 (0.61-0.87)	0.00052	**0.0047**	0.21 (0.12-0.34)	0.95 (0.91-0.98)	0.63	0.049	0.99
AHRI	all	0.55 (0.38-0.73)	0.47	1	1.00 (0.54-1.00)	0.81 (0.70-0.89)	0.4	0.032	0.98
	distal	0.58 (0.39-0.78)	0.35	1	0.11 (0.04-0.24)	0.70 (0.50-0.86)	0.46	0.036	0.99
	proximate	0.81 (0.60-1.00)	0.0077	**0.046**	1.00 (0.40-1.00)	0.95 (0.87-0.99)	0.71	0.055	0.99
MAK	all	0.58 (0.43-0.73)	0.3	1	0.32 (0.16-0.52)	0.82 (0.70-0.91)	0.32	0.025	0.98
	distal	0.54 (0.37-0.71)	0.63	1	0.21 (0.10-0.37)	0.87 (0.72-0.96)	0.29	0.023	0.98
	proximate	0.68 (0.42-0.94)	0.18	0.91	0.11 (0.04-0.23)	1.00 (0.86-1.00)	0.4	0.032	0.98
TOT	all	0.67 (0.62-0.72)	1e-11	**1.6e-10**	0.41 (0.35-0.48)	0.83 (0.80-0.86)	0.51	0.04	0.99
	distal	0.64 (0.59-0.70)	2.4e-07	**2.9e-06**	0.35 (0.28-0.43)	0.86 (0.82-0.88)	0.47	0.037	0.99
	proximate	0.76 (0.68-0.84)	2.8e-08	**3.6e-07**	0.16 (0.11-0.23)	0.97 (0.95-0.98)	0.63	0.049	0.99

Supplementary Table 14. Performance of the TB-ORD model with 450 features when applied to GC6 data. *AUC*, area under curve; *CI*, 95% confidence interval for the AUC; *p-value*, p-value for the AUC; *q-value*, p-value corrected using the Benjamini-Hochberg method; *PPV*, positive predictive value for optimal threshold; *NPV*, negative predictive value; *SENS*, sensitivity at 0.75 specificity; *PPV*^{2} and *NPV*^{2}: PPV and NPV assuming 2% prevalence and at 0.75 sensitivity.

Site	Model	AUC	p-value	q-value	PPV	NPV	SENS	PPV^2	NPV^2
SUN	all	0.67 (0.60-0.74)	4.2e-06	**5.5e-05**	0.33 (0.26-0.40)	0.86 (0.79-0.91)	0.46	0.036	0.99
	distal	0.64 (0.57-0.71)	0.00031	**0.0036**	0.27 (0.21-0.33)	0.90 (0.82-0.95)	0.41	0.032	0.98
	proximate	0.76 (0.57-0.94)	0.0063	**0.044**	0.38 (0.14-0.68)	0.98 (0.95-0.99)	0.6	0.047	0.99
MRC	all	0.63 (0.55-0.72)	0.002	**0.018**	0.44 (0.30-0.59)	0.81 (0.74-0.86)	0.43	0.034	0.98
	distal	0.57 (0.47-0.66)	0.18	0.54	0.21 (0.15-0.29)	0.88 (0.79-0.94)	0.33	0.026	0.98
	proximate	0.73 (0.59-0.88)	0.00075	**0.0075**	0.19 (0.11-0.30)	0.96 (0.92-0.99)	0.63	0.049	0.99
AHRI	all	0.56 (0.38-0.74)	0.42	0.85	0.50 (0.25-0.75)	0.81 (0.69-0.90)	0.45	0.035	0.99
	distal	0.64 (0.44-0.83)	0.13	0.52	0.13 (0.06-0.24)	0.55 (0.23-0.83)	0.49	0.038	0.99
	proximate	0.92 (0.79-1.00)	3e-04	**0.0036**	0.75 (0.35-0.97)	0.98 (0.91-1.00)	0.86	0.065	1
MAK	all	0.62 (0.48-0.77)	0.1	0.5	0.67 (0.30-0.93)	0.83 (0.73-0.91)	0.37	0.029	0.98
	distal	0.48 (0.33-0.64)	0.85	0.85	0.14 (0.05-0.28)	0.78 (0.62-0.90)	0.071	0.0058	0.98
	proximate	0.89 (0.74-1.00)	0.0042	**0.033**	0.57 (0.18-0.90)	0.98 (0.92-1.00)	0.8	0.061	0.99
TOT	all	0.63 (0.58-0.67)	2.9e-07	**4e-06**	0.68 (0.55-0.80)	0.80 (0.76-0.82)	0.41	0.032	0.98
	distal	0.57 (0.52-0.63)	0.0069	**0.044**	0.29 (0.22-0.38)	0.83 (0.79-0.86)	0.35	0.028	0.98
	proximate	0.83 (0.75-0.91)	1.1e-12	**1.7e-11**	0.21 (0.15-0.29)	0.98 (0.96-0.99)	0.76	0.058	0.99

Supplementary Table 15. Q-values (p-values corrected using the Benjamini-Hochberg method) in the differential metabolite analysis. The table shows only the identified metabolites which achieved a q-value lower than 0.01 in one of the two models tested when comparing progressors with controls. *Proximate*, model including only proximate samples (collected within 5 months of TB

diagnosis); *Full*, model including all samples. Enries show odds ratios (*OR*) with 95% confidence intervals and q values corrected for multiple testing using the Benjamini-Hochberg method.

ID	Name	Group	Proximate	q	Full	q
M.42374	2-aminobutyrate	Amino Acid	0.10 (0.02-0.39)	**0.0049**	1.31 (0.83-2.08)	0.48
M.36746	2-hydroxy-3-methylvalerate	„	1.28 (0.90-1.83)	0.32	1.35 (1.14-1.61)	**0.0016**
M.21044	2-hydroxybutyrate (AHB)	„	0.90 (0.42-1.92)	0.87	1.60 (1.22-2.09)	**0.0058**
M.44618	3-methoxytyramine sulfate	„	1.82 (1.12-2.94)	**0.0089**	1.33 (1.06-1.67)	0.051
M.12017	3-methoxytyrosine	„	166.86 (14.76-1886.48)	**6.8e-05**	1.85 (0.86-3.97)	0.29
M.32349	4-imidazoleacetate	„	2.11 (1.48-3.00)	**6.8e-05**	1.14 (1.00-1.31)	0.18
M.1126	alanine	„	0.05 (0.01-0.30)	**0.0042**	0.16 (0.07-0.35)	**7.7e-05**
M.12129	beta-hydroxyisovalerate	„	3.36 (1.61-7.04)	**0.0089**	2.44 (1.69-3.51)	**7.7e-05**
M.32675	C-glycosyltryptophan	„	3.40 (1.62-7.12)	**0.0038**	1.69 (1.20-2.38)	**0.013**
M.31453	cysteine	„	0.63 (0.32-1.26)	0.31	0.50 (0.33-0.75)	**0.0041**
M.53	glutamine	„	0.13 (0.03-0.62)	**0.032**	0.29 (0.14-0.61)	**0.0079**
M.43802	guanidinoacetate	„	0.17 (0.06-0.51)	**0.0078**	0.51 (0.32-0.82)	**0.03**
M.59	histidine	„	**0.02 (0.00-0.12)**	**0.00017**	0.12 (0.05-0.29)	**7.7e-05**
M.1302	methionine	„	0.07 (0.02-0.31)	**0.0018**	0.29 (0.15-0.58)	**0.0033**
M.43488	N-acetylcarnosine	„	0.30 (0.12-0.73)	**0.027**	0.39 (0.26-0.59)	**7.7e-05**
M.27710	N-acetylglycine	„	0.94 (0.56-1.59)	0.89	1.54 (1.28-1.87)	**0.00021**
M.1589	N-acetylmethionine	„	4.02 (2.06-7.82)	**0.00029**	1.22 (0.93-1.60)	0.34
M.33950	N-acetylphenylalanine	„	2.58 (1.48-4.49)	**0.0089**	1.67 (1.30-2.16)	**0.00089**

M.1444	pipecolate	„	1.18 (1.05-1.33)	**0.027**	1.11 (1.04-1.19)	**0.0064**
M.2125	taurine	„	4.84 (2.04-11.51)	**0.0042**	1.35 (0.86-2.11)	0.4
M.1284	threonine	„	0.07 (0.01-0.36)	**0.0063**	0.49 (0.24-0.99)	0.15
M.54	tryptophan	„	**0.02 (0.00-0.16)**	**0.00087**	0.11 (0.04-0.30)	**0.00022**
M.47709	2-oxoarginine	„	1.10 (0.84-1.45)	0.66	0.72 (0.58-0.89)	**0.0076**
M.47715	argininate	„	0.77 (0.43-1.36)	0.53	0.57 (0.40-0.80)	**0.0048**
M.46908	6-bromotryptophan	„	0.07 (0.02-0.29)	**0.00072**	0.53 (0.32-0.90)	0.07
M.46928	valine ion	„	0.09 (0.01-0.91)	0.13	0.12 (0.04-0.38)	**0.0041**
M.584	mannose	Carbohydrate	2.30 (0.99-5.35)	0.17	3.15 (2.04-4.87)	**1.2e-05**
M.42582	pyruvate	„	2.29 (1.42-3.69)	**0.0083**	1.10 (0.92-1.32)	0.53
M.15053	sorbitol	„	1.68 (1.24-2.27)	**0.00072**	1.16 (1.07-1.27)	**0.00043**
M.27738	threonate	Cofactors and Vitamins	1.00 (0.71-1.41)	1	0.69 (0.56-0.86)	**0.0044**
M.33453	alpha-ketoglutarate	Energy	2.47 (1.49-4.08)	**0.0033**	1.28 (1.03-1.57)	0.11
M.1564	citrate	„	**0.04 (0.01-0.21)**	**0.00059**	0.31 (0.17-0.58)	**0.0026**
M.39609	16-hydroxypalmitate	Lipid and lipid-like molecules	1.04 (0.54-2.00)	0.94	1.70 (1.25-2.32)	**0.0067**
M.44630	1-dihomo-linolenoyl-GPE (20:3n3 or 6)	„	0.65 (0.34-1.26)	0.35	0.57 (0.40-0.79)	**0.0058**
M.27447	1-linoleoylglycerol (18:2)	„	0.22 (0.10-0.50)	**2.2e-05**	0.87 (0.76-1.01)	0.14
M.36594	1-linoleoyl-GPI (18:2)	„	1.83 (1.08-3.09)	0.096	1.56 (1.23-1.99)	**0.004**
M.21184	1-oleoylglycerol (18:1)	„	0.47 (0.28-0.78)	**0.0032**	0.98 (0.90-1.06)	0.71
M.44621	1-(1-enyl-oleoyl)-GPE (P-18:1)	„	1.50 (0.97-2.32)	0.19	1.47 (1.17-1.86)	**0.009**

M.42489	2-hydroxydecanoate	„	0.21 (0.08-0.52)	**0.00072**	0.64 (0.48-0.87)	**0.0051**
M.37253	2-hydroxyglutarate	„	2.63 (1.44-4.80)	**0.013**	1.63 (1.21-2.19)	**0.0031**
M.542	3-hydroxybutyrate (BHBA)	„	0.93 (0.76-1.14)	0.64	1.09 (1.04-1.15)	**0.0027**
M.37202	androstenediol (3beta,17beta) disulfate (1)	„	1.32 (1.05-1.65)	0.053	1.17 (1.07-1.27)	**0.0015**
M.37187	5alpha-androstan-3beta,17alpha-diol disulfate	„	1.07 (0.90-1.26)	0.64	1.19 (1.09-1.30)	**0.0017**
M.37190	5alpha-androstan-3beta,17beta-diol disulfate	„	1.16 (0.99-1.36)	0.18	1.21 (1.12-1.31)	**9.6e-07**
M.1712	cortisol	„	8.42 (3.29-21.57)	**9.5e-05**	3.16 (2.04-4.89)	**1.7e-05**
M.1769	cortisone	„	6.10 (1.66-22.44)	**0.034**	2.92 (1.54-5.50)	**0.0078**
M.32599	glycocholenate sulfate	„	6.90 (3.11-15.30)	**2.7e-06**	2.03 (1.47-2.79)	**0.00017**
M.42574	glycohyocholate	„	1.31 (1.10-1.56)	**0.00045**	1.12 (1.03-1.21)	**0.0067**
M.43264	3-hydroxybutyrylcarnitine (1)	„	0.88 (0.51-1.53)	0.78	1.21 (1.07-1.37)	**0.0094**
M.46739	laurylcarnitine (C12)	„	1.02 (0.76-1.37)	0.93	1.22 (1.09-1.38)	**0.0068**
M.34035	linolenate [alpha or gamma; (18:3n3 or 6)]	„	0.48 (0.29-0.80)	**0.0078**	0.98 (0.85-1.13)	0.9
M.33952	myristoylcarnitine (C14)	„	1.37 (0.96-1.95)	0.23	1.47 (1.23-1.74)	**2e-04**
M.38102	oleoyl ethanolamide	„	2.97 (1.55-5.70)	**0.011**	1.95 (1.35-2.82)	**0.0046**
M.35160	oleoylcarnitine (C18:1)	„	1.87 (1.19-2.94)	**0.04**	1.59 (1.24-2.02)	**0.0026**
M.32562	pregnenediol disulfate (C21H34O8S2)	„	2.49 (1.59-3.88)	**6e-04**	1.54 (1.25-1.89)	**7e-04**
M.34445	sphingosine 1-phosphate	„	3.98 (2.04-7.79)	**0.00072**	1.91 (1.37-2.65)	**0.0016**
M.34409	stearoylcarnitine (C18)	„	1.81 (1.09-2.99)	0.097	1.77 (1.35-2.31)	**0.00059**
M.32807	taurocholenate sulfate	„	2.40 (1.63-3.54)	**9.5e-05**	1.31 (1.10-1.55)	**0.016**

M.46500	3-CMPFP	„	0.11 (0.04-0.33)	**6.8e-05**	0.55 (0.39-0.79)	**0.0053**
M.46670	3,4-methylene heptanoyl carnitine	„	0.32 (0.16-0.64)	**0.0016**	0.92 (0.76-1.11)	0.59
M.46471	hexanoylglutamine	„	0.97 (0.74-1.28)	0.91	1.15 (1.06-1.25)	**0.0017**
M.46751	3-hydroxybutyrylcarnitine (2)	„	1.11 (0.72-1.69)	0.78	1.45 (1.21-1.72)	**0.00053**
M.46660	myristoleoylcarnitine	„	1.06 (0.84-1.33)	0.78	1.18 (1.07-1.30)	**0.0046**
M.47640	androsterone glucuronide	„	0.23 (0.11-0.48)	**6.8e-05**	0.77 (0.64-0.94)	**0.037**
M.1559	5,6-dihydrouracil	Nucleotide	14.93 (4.02-55.41)	**0.00031**	1.90 (1.08-3.35)	0.094
M.35157	N6-carbamoylthreonyladenosine	„	14.24 (3.14-64.52)	**0.0039**	1.89 (1.13-3.19)	0.065
M.47391	2'-O-methyluridine	„	1.24 (0.83-1.85)	0.5	1.87 (1.46-2.41)	**4.1e-05**
M.37104	cyclo(leu-pro)	Peptide	0.58 (0.36-0.94)	**0.024**	0.76 (0.64-0.90)	**0.0022**
M.2730	gamma-glutamylglutamine	„	0.39 (0.17-0.90)	0.095	0.42 (0.26-0.66)	**0.002**
M.34456	gamma-glutamylisoleucine	„	2.07 (1.42-3.01)	**6e-04**	1.50 (1.26-1.80)	**0.00014**
M.18369	gamma-glutamylleucine	„	2.42 (1.52-3.84)	**0.00087**	1.61 (1.27-2.05)	**0.0015**
M.33422	gamma-glutamylphenylalanine	„	8.11 (2.88-22.84)	**0.00085**	2.51 (1.50-4.18)	**0.0047**
M.43829	gamma-glutamylvaline	„	1.84 (1.34-2.52)	**0.00072**	1.38 (1.18-1.62)	**0.00053**
M.32836	HWESASXX	„	1.43 (1.16-1.77)	**0.0018**	1.06 (0.99-1.15)	0.27
M.38002	1,2-propanediol	Xenobiotics	1.47 (1.06-2.04)	0.092	1.21 (1.08-1.36)	**3e-04**
M.33173	2-hydroxyacetaminophen sulfate	„	0.46 (0.06-3.57)	0.41	1.11 (1.03-1.19)	**0.0028**
M.43400	2-piperidinone	„	1.42 (1.16-1.74)	**0.00072**	1.14 (1.05-1.24)	**0.0017**
M.553	cotinine	„	0.89 (0.37-2.14)	0.88	1.49 (1.17-1.91)	**0.0076**

M.33935	piperine	„	0.76 (0.57-1.01)	**0.035**	0.83 (0.74-0.93)	**9e-04**
M.20693	tartronate (hydroxymalonate)	„	0.52 (0.32-0.85)	**0.025**	0.71 (0.57-0.87)	**0.0068**
M.46622	thioproline	„	0.33 (0.15-0.71)	**0.017**	0.46 (0.31-0.67)	**0.00059**
M.47986	eugenol sulfate	„	0.93 (0.82-1.04)	0.13	0.93 (0.88-0.98)	**0.0016**

Supplementary Table 16. Significant ($q < 0.001$) results of the regression in progressors over time to diagnosis. *ΔBIC*, difference in Bayesian Information Criterion between the null model and the full model including time to TB; f^2, Cohen's f^2 effect size measure; *q*, p-value of likelihood ratio test corrected for multiple testing using the Benjamini-Hochberg method.

ID	Name	Group	ΔBIC	f^2	q
M.33950	N-acetylphenylalanine	Amino Acid	63	0.46	9.1e-14
M.64	phenylalanine	„	32	0.23	1.2e-07
M.46473	1-carboxyethylphenylalanine	„	26	0.19	1.9e-06
M.36752	N6-acetyllysine	„	22	0.16	7.1e-06
M.32390	N-acetyltyrosine	„	22	0.16	7.1e-06
M.15140	kynurenine	„	20	0.15	2e-05
M.1587	N-acetylleucine	„	19	0.14	3.1e-05
M.43231	6-oxopiperidine-2-carboxylate	„	17	0.13	6.7e-05
M.1299	tyrosine	„	16	0.12	8.3e-05
M.43802	guanidinoacetate	„	15	0.12	0.00012
M.15681	4-guanidinobutanoate	„	14	0.11	0.00017
M.33939	N-acetylthreonine	„	14	0.11	0.00018
M.32197	3-(4-hydroxyphenyl)lactate	„	13	0.11	0.00029
M.33967	N-acetylisoleucine	„	12	0.1	0.00041
M.6146	2-aminoadipate	„	11	0.092	0.00071
M.60	leucine	„	10	0.09	8e-04
M.527	lactate	Carbohydrate	18	0.14	4e-05
M.33477	erythronate	„	13	0.11	0.00027
M.20488	glucose	„	12	0.098	0.00048
M.32586	bilirubin (E,E)	Cofactors and Vitamins	29	0.21	5.5e-07
M.43807	bilirubin (Z,Z)	„	11	0.095	0.00058

M.1712	cortisol	Lipid	23	0.17	5.6e-06
M.32807	taurocholenate sulfate	„	23	0.17	5.7e-06
M.18476	glycocholate	„	23	0.17	5.8e-06
M.18494	taurochenodeoxycholate	„	23	0.17	5.8e-06
M.32346	glycochenodeoxycholate	„	19	0.15	2.7e-05
M.32418	myristoleate (14:1n5)	„	19	0.14	3e-05
M.15122	glycerol	„	18	0.14	4.2e-05
M.18497	taurocholate	„	17	0.13	6e-05
M.22053	3-hydroxydecanoate	„	17	0.13	6.2e-05
M.46388	dodecadienoate (12:2)	„	17	0.13	6.2e-05
M.34035	linolenate [alpha or gamma; (18:3n3 or 6)]	„	16	0.13	7.6e-05
M.32425	dehydroisoandrosterone sulfate (DHEA-S)	„	15	0.12	0.00012
M.33936	octanoylcarnitine (C8)	„	13	0.11	0.00033
M.18477	glycodeoxycholate	„	13	0.1	0.00034
M.1336	palmitate (16:0)	„	13	0.1	0.00034
M.19324	1-stearoyl-GPI (18:0)	„	12	0.1	0.00036
M.33941	decanoylcarnitine (C10)	„	12	0.1	0.00036
M.33973	epiandrosterone sulfate	„	12	0.1	0.00036
M.47640	androsterone glucuronide	„	12	0.099	0.00045
M.1365	myristate (14:0)	„	12	0.099	0.00047
M.37418	1-pentadecanoyl-GPC (15:0)	„	12	0.098	0.00048
M.1105	linoleate (18:2n6)	„	11	0.097	0.00051
M.32497	10-undecenoate (11:1n1)	„	11	0.095	0.00058
M.1645	laurate (12:0)	„	11	0.092	0.00073
M.32328	hexanoylcarnitine (C6)	„	10	0.09	8e-04
M.36850	taurolithocholate 3-sulfate	„	10	0.09	8e-04

M.46500	3-CMPFP	„	10	0.089	0.00086
M.39379	glycoursodeoxycholate	„	10	0.088	0.00088
M.1107	allantoin	Nucleotide	44	0.31	9.2e-10
M.35157	N6-carbamoylthreonyladenosine	„	24	0.17	4.7e-06
M.35137	N2,N2-dimethylguanosine	„	18	0.14	4.3e-05
M.47608	N6-succinyladenosine	„	13	0.1	0.00033
M.1559	5,6-dihydrouracil	„	11	0.091	0.00073
M.15136	xanthosine	„	10	0.089	0.00083
M.33422	gamma-glutamylphenylalanine	Peptide	38	0.27	1e-08
M.2734	gamma-glutamyltyrosine	„	17	0.13	6.7e-05
M.18369	gamma-glutamylleucine	„	15	0.12	0.00015
M.34456	gamma-glutamylisoleucine	„	11	0.091	0.00073

Supplementary Table 17. Results of the enrichment analysis for the time course regression. ID, identifier of the cluster or metabolite set; Name, name of the cluster or metabolite set; *AUC*, area under curve; *q*, p-value for the U statistic of the AUC adjusted for multiple testing using the Benjamini-Hochberg method.

ID	Name	AUC	q
ME.2	Long chain fatty acid cluster	0.77	**5.6e-05**
ME.37	Kynurenines, taurocholates and cortisol cluster	0.81	**5.6e-05**
MP.1	Lipid	0.64	**0.00095**
MS.36	Long Chain Fatty Acid	0.72	**0.021**
MS.40	Polyunsaturated Fatty Acid (n3 and n6)	0.65	0.12
MS.1	Steroid	0.65	0.16
MS.2	Phenylalanine and Tyrosine Metabolism	0.59	0.26
MS.37	Medium Chain Fatty Acid	0.57	0.33
MP.2	Amino Acid	0.52	0.39

Supplementary Table 18. Detailed information on the computing environment used to perform the analyses (sessionInfo() output).

```
## R version 3.4.4 (2018-03-15)
## Platform: x86_64-pc-linux-gnu (64-bit)
## Running under: Ubuntu 16.04.5 LTS
##
## Matrix products: default
## BLAS: /usr/lib/libblas/libblas.so.3.6.0
## LAPACK: /usr/lib/lapack/liblapack.so.3.6.0
##
## locale:
##  [1] LC_CTYPE=en_US.UTF-8          LC_NUMERIC=C
##  [3] LC_TIME=en_US.UTF-8           LC_COLLATE=en_US.UTF-8
##  [5] LC_MONETARY=en_US.UTF-8       LC_MESSAGES=en_US.UTF-8
##  [7] LC_PAPER=en_US.UTF-8          LC_NAME=en_US.UTF-8
##  [9] LC_ADDRESS=en_US.UTF-8        LC_TELEPHONE=en_US.UTF-8
## [11] LC_MEASUREMENT=en_US.UTF-8    LC_IDENTIFICATION=en_US.UTF-8
##
## attached base packages:
## [1] stats     graphics  grDevices utils     datasets  methods   base
##
## other attached packages:
##  [1] xlsx_0.6.1             lme4_1.1-17            Matrix_1.2-14
##  [4] epiR_0.9-96            survival_2.42-6        tmod_0.39
##  [7] sm_2.2-5.5             segmented_0.5-3.0      randomForest_4.6-14
## [10] pROC_1.12.1            plotwidgets_0.5        pander_0.6.2
## [13] OptimalCutpoints_1.1-3 limma_3.30.13          gplots_3.0.1
## [16] beeswarm_0.2.3         rmarkdown_1.10         pca3d_0.10
## [19] myfuncs_1.8            setwidth_1.0-4         colorout_1.1-2
##
## loaded via a namespace (and not attached):
##   [1] tagcloud_0.6           minqa_1.2.4
##   [3] colorspace_1.3-2       class_7.3-14
##   [5] rprojroot_1.3-2        pls_2.6-0
##   [7] DRR_0.0.3              prodlim_2018.04.18
##   [9] lubridate_1.7.4        codetools_0.2-15
##  [11] splines_3.4.4          robustbase_0.93-2
##  [13] knitr_1.20             RcppRoll_0.3.0
##  [15] jsonlite_1.5           nloptr_1.0.4
##  [17] caret_6.0-80           rJava_0.9-10
##  [19] broom_0.5.0            ddalpha_1.3.4
##  [21] kernlab_0.9-27         sfsmisc_1.1-2
##  [23] shiny_1.1.0            compiler_3.4.4
##  [25] backports_1.1.2        assertthat_0.2.0
##  [27] lazyeval_0.2.1         later_0.7.3
##  [29] htmltools_0.3.6        tools_3.4.4
##  [31] bindrcpp_0.2.2         gtable_0.2.0
##  [33] glue_1.3.0             reshape2_1.4.3
##  [35] dplyr_0.7.6            Rcpp_0.12.18
##  [37] gdata_2.18.0           nlme_3.1-137
```

```
## [39] iterators_1.0.10        crosstalk_1.0.0
## [41] timeDate_3043.102       gower_0.1.2
## [43] stringr_1.3.1           xlsxjars_0.6.1
## [45] mime_0.5                miniUI_0.1.1.1
## [47] gtools_3.8.1            XML_3.98-1.15
## [49] DEoptimR_1.0-8          MASS_7.3-50
## [51] scales_1.0.0            ipred_0.9-7
## [53] promises_1.0.1          RColorBrewer_1.1-2
## [55] yaml_2.2.0              ggplot2_3.0.0
## [57] rpart_4.1-13            stringi_1.2.4
## [59] foreach_1.4.4           caTools_1.17.1.1
## [61] manipulateWidget_0.10.0 lava_1.6.3
## [63] geometry_0.3-6          rlang_0.2.1
## [65] pkgconfig_2.0.1         bitops_1.0-6
## [67] rgl_0.99.16             evaluate_0.11
## [69] lattice_0.20-35         purrr_0.2.5
## [71] bindr_0.1.1             recipes_0.1.3
## [73] htmlwidgets_1.2         CVST_0.2-2
## [75] tidyselect_0.2.4        plyr_1.8.4
## [77] magrittr_1.5            R6_2.2.2
## [79] dimRed_0.1.0            pillar_1.3.0
## [81] withr_2.1.2             abind_1.4-5
## [83] nnet_7.3-12             tibble_1.4.2
## [85] crayon_1.3.4            KernSmooth_2.23-15
## [87] ellipse_0.4.1           grid_3.4.4
## [89] data.table_1.11.4       ModelMetrics_1.2.0
## [91] digest_0.6.15           webshot_0.5.0
## [93] xtable_1.8-2            tidyr_0.8.1
## [95] httpuv_1.4.5            stats4_3.4.4
## [97] munsell_0.5.0           BiasedUrn_1.07
## [99] magic_1.5-8             tcltk_3.4.4
```
