# Supplementary material for: Metabolite changes in blood predict the onset of tuberculosis
Source: Nat Commun. 2018 Dec 6;9:5208. doi: 10.1038/s41467-018-07635-7 (PMC6283869; doi:10.1038/s41467-018-07635-7)
Supplement: Supplementary file 3 — Reporting Summary [file 41467_2018_7635_MOESM3_ESM.pdf]

nature research

Corresponding author(s): Stefan H. E. Kaufmann

Reporting Summary

Nature Research wishes to improve the reproducibility of the work that we publish. This form provides structure for consistency and transparency in reporting. For further information on Nature Research policies, see Authors & Referees and the Editorial Policy Checklist.

Statistical parameters

When statistical analyses are reported, confirm that the following items are present in the relevant location (e.g. figure legend, table legend, main text, or Methods section).

n/a	Confirmed	
☐	☒	The exact sample size (*n*) for each experimental group/condition, given as a discrete number and unit of measurement
☐	☒	An indication of whether measurements were taken from distinct samples or whether the same sample was measured repeatedly
☐	☒	The statistical test(s) used AND whether they are one- or two-sided<br>*Only common tests should be described solely by name; describe more complex techniques in the Methods section.*
☐	☒	A description of all covariates tested
☐	☒	A description of any assumptions or corrections, such as tests of normality and adjustment for multiple comparisons
☐	☒	A full description of the statistics including central tendency (e.g. means) or other basic estimates (e.g. regression coefficient) AND variation (e.g. standard deviation) or associated estimates of uncertainty (e.g. confidence intervals)
☐	☒	For null hypothesis testing, the test statistic (e.g. *F*, *t*, *r*) with confidence intervals, effect sizes, degrees of freedom and *P* value noted<br>*Give P values as exact values whenever suitable.*
☒	☐	For Bayesian analysis, information on the choice of priors and Markov chain Monte Carlo settings
☐	☒	For hierarchical and complex designs, identification of the appropriate level for tests and full reporting of outcomes
☐	☒	Estimates of effect sizes (e.g. Cohen's *d*, Pearson's *r*), indicating how they were calculated
☐	☒	Clearly defined error bars<br>*State explicitly what error bars represent (e.g. SD, SE, CI)*

Our web collection on statistics for biologists may be useful.

Software and code

Policy information about availability of computer code

Data collection	Only hardware drivers (i.e., software for operating the mass spectrometry analysis hardware) was used in data collection.
Data analysis	All data analysis was performed using the R computing platform. The manuscript has been prepared as an Rmarkdown document, therefore full accountability of methods used is guaranteed. Full list of dependencies, libraries used and their versions used in manuscript preparation are shown on Supplementary Table 17. All generic code and all computational steps have been made available to the reviewers and to the readers upon publication. The code and source data can be accessed at github.com/january3/gc6metabolomics.

For manuscripts utilizing custom algorithms or software that are central to the research but not yet described in published literature, software must be made available to editors/reviewers upon request. We strongly encourage code deposition in a community repository (e.g. GitHub). See the Nature Research guidelines for submitting code & software for further information.

Data

Policy information about availability of data

All manuscripts must include a data availability statement. This statement should provide the following information, where applicable:

- Accession codes, unique identifiers, or web links for publicly available datasets
- A list of figures that have associated raw data
- A description of any restrictions on data availability

All data are accessible to the readers using the Metabolomic Workbench platform upon the publication of the manuscript (Project ID PR000666). In addition, all data ad meta data necessary to replicate the findings and the figures are available from github.com/january3/gc6metabolomics.

Field-specific reporting

Please select the best fit for your research. If you are not sure, read the appropriate sections before making your selection.

☒ Life sciences ☐ Behavioural & social sciences ☐ Ecological, evolutionary & environmental sciences

For a reference copy of the document with all sections, see nature.com/authors/policies/ReportingSummary-flat.pdf

Life sciences study design

All studies must disclose on these points even when the disclosure is negative.

Sample size	Cohort size was determined a priori by calculating the expected number of progressors (individuals who develop TB during the study) and the known prevalence of TB.
Data exclusions	Samples from HIV+ individuals were excluded from the study due to (i) lower number and (ii) to avoid confounding effects. Low QC samples were also excluded from the study (See consort diagram, Fig. 1 in the manuscript).
Replication	Additional, external data from TB patients and two other groups (healthy individuals and other respiratory diseases) was used to validate the key findings.
Randomization	N/A (this was a longitudinal prospective study)
Blinding	The initial data was split into a training (2/3) and test set (1/3). The computational biologists (January Weiner and Fergal Duffy) who were analysing the data were blinded for the test set. The unblinding information was sent out only after the blinded predictions for the test set have been disseminated to the GC6 consortium.

Reporting for specific materials, systems and methods

Materials & experimental systems

n/a	Involved in the study
☒	☐ Unique biological materials
☒	☐ Antibodies
☒	☐ Eukaryotic cell lines
☒	☐ Palaeontology
☒	☐ Animals and other organisms
☐	☒ Human research participants

Methods

n/a	Involved in the study
☒	☐ ChIP-seq
☒	☐ Flow cytometry
☒	☐ MRI-based neuroimaging

Human research participants

Policy information about studies involving human research participants

Population characteristics	Detailed information on all samples and all patients (such as gender and age) is available in the supplementary data disseminated at the URL specified in the Methods section at present and have been made available as meta-data through Metabolomics Workbench (PR000666).
Recruitment	We recruited household contacts of TB index cases who were diagnosed at one of the four centres involved in the study. This is described in Methods, "Study design and participants".
